# Guiding AlphaFold predictions with experimental knowledge to inform dynamics and interactions with VAIRO

**DOI:** 10.1002/pro.70481

**Published:** 2026-01-24

**Authors:** Josep Triviño, Elisabet Jiménez, Christoph Grininger, Iracema Caballero, Ana Medina, Albert Castellví, Giovanna Petrillo, Fernando Govantes, Theo Sagmeister, Martín Alcorlo, Juan A. Hermoso, Massimo D. Sammito, Kay Diederichs, Tea Pavkov‐Keller, Isabel Usón

**Affiliations:** ^1^ Department of Structural Biology Instituto de Biología Molecular de Barcelona (IBMB‐CSIC) Barcelona Spain; ^2^ Facultad de Farmacia Universidad de Barcelona Barcelona Spain; ^3^ Institute of Molecular Biosciences University of Graz Graz Austria; ^4^ Centro Andaluz de Biología del Desarrollo Universidad Pablo de Olavide/Consejo Superior de Investigaciones Científicas/Junta de Andalucía Sevilla Spain; ^5^ Departamento de Biología Molecular e Ingeniería Bioquímica Universidad Pablo de Olavide Sevilla Spain; ^6^ Department of Crystallography and Structural Biology Institute of Physical‐Chemistry Blas Cabrera (IQF‐CSIC) Madrid Spain; ^7^ Fachbereich Biologie Universität Konstanz Constance Germany; ^8^ Field of Excellence BioHealth University of Graz Graz Austria; ^9^ BioTechMed‐Graz Graz Austria; ^10^ ICREA: Institució Catalana de Recerca i Estudis Avançats Barcelona Spain

**Keywords:** ABC transporter, AlphaFold, bacterial surface layer, dynamics, interactions, predictions, structure, VAIRO

## Abstract

Structural predictions have reached unprecedented accuracy. They leverage sequence‐specific data to capture all potential interactions a sequence has evolved to fulfill. AlphaFold derives information from three sources: learned parameters capturing intrinsic amino acid secondary structure and environment propensity; models of related proteins providing structural templates; and aligned sequences encoding profiles and concerted evolutionary changes of residues involved in contacts. However, function demands dynamic changes; hence not all possible interactions can coexist simultaneously. Comprehensive information entails contradictions, which resolved in favor of the better‐informed structure will silence less stable states and associations. Here, we introduce a method using all three channels to include prior knowledge: site‐specific variants, predefined alignments and templates. Selecting information relevant to a particular state delimits the functional context of a prediction. Our program VAIRO allows us to rescue asymmetric and weaker interactions to complete the view of molecular assemblies in the architecture of a bacterial surface layer, and reveals otherwise inaccessible dynamic states in a pneumococcal multimeric membrane protein complex. VAIRO is distributed via the python package index (PyPI) (https://pypi.org/project/vairo) and the code is also available on Github (https://github.com/arcimboldo-team/vairo).

## 1 INTRODUCTION

AlphaFold (AF) (Jumper et al., 2021) has advanced most sequence‐based structure predictions to the accuracy of close homologs, as assessed in CASP14‐15 experiments (Kryshtafovych et al., 2019). Along with RoseTTAFold (Baek et al., 2021), these tools have spread throughout structural biology enhancing our view beyond the available experimental structures. AF predictions are now broadly accessible through ColabFold (Mirdita et al., 2022), and databases of precomputed monomeric predictions for genomic sequences in model organisms (Varadi et al., 2022). AF application has been extended to multimer prediction (Evans et al., 2022), in favorable cases reaching comparable success to state‐of‐the‐art docking or fold and dock methods (Bryant et al., 2022) and enhancing our view of whole interactomes (Abramson et al., 2024; Durairaj et al., 2023; Humphreys et al., 2021).

Porting the powerful attention algorithm (Vaswani et al., 2017) from the BERT Transformer (Devlin et al., 2019), originally developed for natural language translation, succeeded in the translation of sequence into three‐dimensional structure. In language translation, the fundamental objective is to apprehend the meaning, rather than merely finding equivalent words. Likewise, the ultimate goal of a structure predictor would be to translate sequence into function, which entails interactions, organization into more complex structures, and dynamics (Banerjee et al., 2023). This structural potential must be enabled by the sequence, but its realization depends on the context. Accordingly, establishing the background by providing consistent prior knowledge to the structure prediction algorithm underlying AF should draw inferences that are otherwise lost in the resolution of contradictory information.

Alternative, retrainable implementations such as OpenFold (Ahdritz et al., 2022) and Uni‐Fold (Li et al., 2022) ease integrating other experimental data sources, as in AlphaLink2 (Stahl et al., 2023, 2024), which exploits crosslinking mass spectrometry in the prediction of complexes. Still, the native information channels in AF can be directly exploited without the need to retrain, giving rise to strategies to enhance AF predictions to cover a broader structural space. For instance, an iterative procedure has been developed in which AF models are automatically rebuilt on experimental electron density maps, and the rebuilt models are then used as templates for new AF predictions (Terwilliger et al., 2022). In other novel approaches, SymProFold (Buhlheller et al., 2024) selects with symmetry boundary conditions AF‐predicted multimers consistent with planar structures in bacterial surface layers or AF_unmasked (Mirabello et al., 2024) leverages information from multimeric templates to build large protein complexes, so that the positioning of protein chains with respect to each other informs the prediction. Likewise, reducing the depth of the input multiple sequence alignments (MSA) has been shown to better sample the conformational landscape of proteins like transporters and receptors in monomeric structures adopting multiple states in a dynamic mechanism (del Alamo et al., 2022). Building on this, AF‐Cluster (Wayment‐Steele et al., 2024) partitions the MSA by sequence similarity and runs AF2 on each cluster separately. Different clusters of homologs bias AF2 to predict different conformations of metamorphic proteins, G protein‐coupled receptors, and kinases (Sala et al., 2023). Similarly, AFsample2 (Kalakoti & Wallner, 2025) applies random masking to MSA columns to reduce co‐evolutionary signals, to force AF to explore alternative conformations. Finally, high‐throughput pipelines like AlphaPulldown2 (Molodenskiy et al., 2025) automate protein–protein interaction screening using AF‐Multimer and retrainable versions of AF.

Here, we present a general method to guide AF predictions, rescuing weaker interactions to reveal otherwise inaccessible dynamic states and complete the architecture of large macromolecular assemblies. We have developed in our program VAIRO (http://chango.ibmb.csic.es/VAIRO) an approach tailored to modify AF's native information search and provide features designed on prior knowledge to set boundary conditions relevant to predicting a particular functional state.

## 2 RESULTS

### 2.1 Establishing method principles: Information sources in AF predictions

AF predictions do not come from the query sequence alone. For each sequence, AF searches large genomic and structural databases (e.g., Protein Data Bank (PDB) [Berman et al., 2000] and UniProt [UniProt, 2023]) to retrieve related information. For this genomic and structural profiling, the original AF distribution utilizes the HH‐suite (Steinegger et al., 2019), whereas the fast ColabFold implementation relies on MMseqs2 (Mirdita et al., 2019).

**FIGURE 1:**
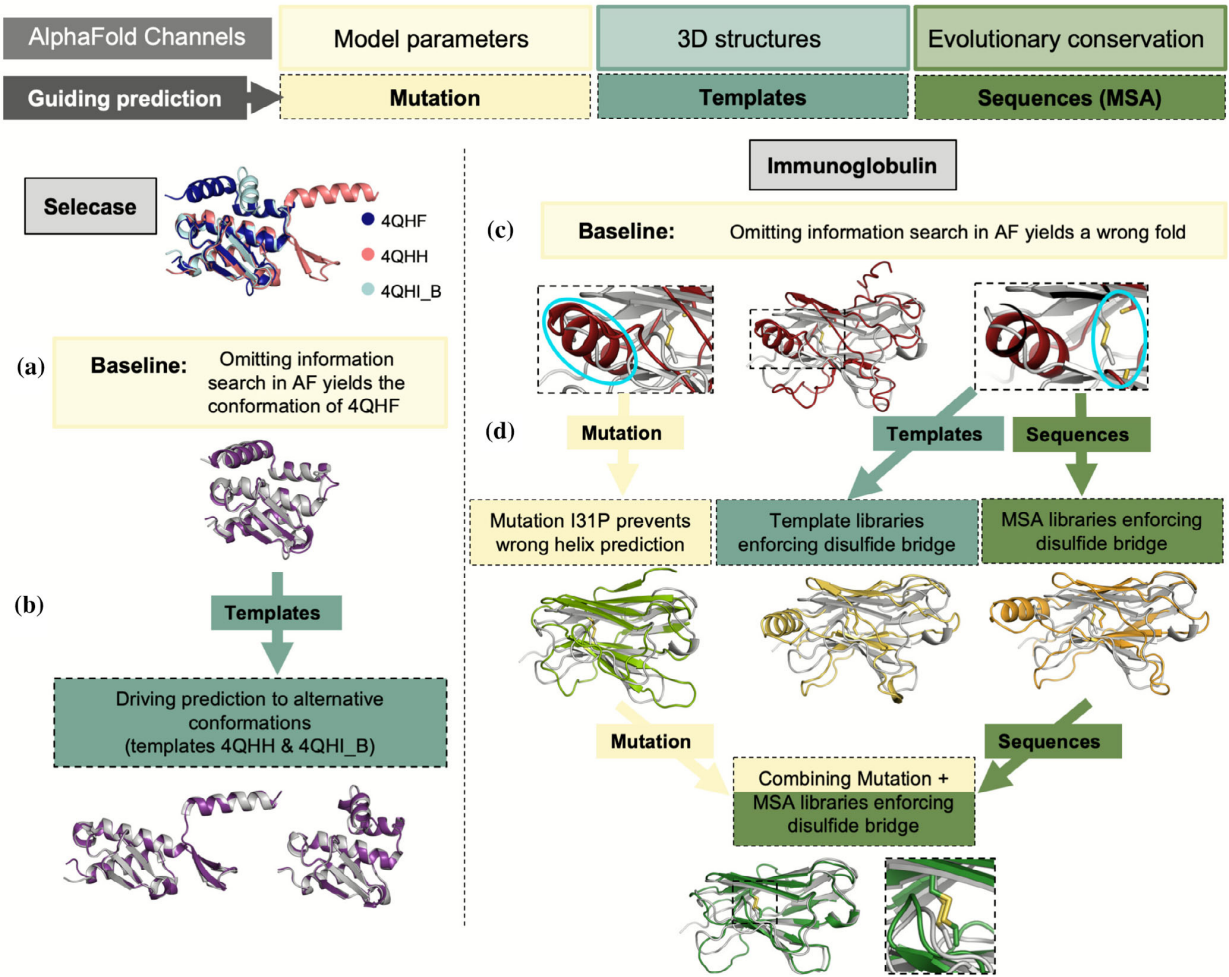
Channels to guide AlphaFold (AF) illustrated on predictions with minimal information and comparison of the predicted models (colored) and experimental structures (gray unless otherwise specified). (a) Selecase baseline prediction (excluding any information beyond its sequence) superposed to the closest one of its known crystal structures. (b) Driving predictions toward its alternative conformations with templates. (c) Immunoglobulin baseline prediction fails to yield the correct fold. The circles highlight the wrong helix and the missing disulfide bridge. (d) Providing additional information to guide the prediction: A point mutation I31P in the query sequence prevents prediction of the wrong helix; promoting the disulfide bridge with a library of models made of two beta strands connected by a disulfide bridge input to match the cysteines and their flanking residues (15 amino acids per model) or alternatively inputting the sequences of each of these models results in disulfide bridge formation and improves the stereochemistry of the beta‐sheets; combining several ways approximates the prediction toward the experimental structure. 3D, three‐dimensional; MAS, multiple sequence alignments.

AF derives its prediction from three sources: template models of related proteins providing structural information; MSAs that supply aligned sequences encoding profiles (Steinegger et al., 2019) and pairwise conservation of residues involved in contacts (Marks et al., 2011); and learned parameters capturing intrinsic amino acid secondary structure and environment propensity. Each channel can be used to steer a prediction: templates can be tailored to provide direct structural information; MSA can be generated, eliminated, or locally masked to promote or hamper interactions; and although modifying the learned weights would require retraining, the query sequence may be mutated to change residue propensities (e.g., introducing prolines to break helices) (Figure 1). The results from the database searches (templates and MSA) aligned to the query sequence are stored in a single file called features.pkl, containing a multidimensional array that steers the prediction.

Our method, VAIRO, enables extensive and flexible customization of AF inputs by modifying the features.pkl, facilitating the prediction of large protein assemblies across different dynamical states. To gain knowledge about the biological system, VAIRO can automatically analyze AF‐search data to identify relevant structural clusters that may represent alternative conformations. AF2 uses OpenMM (Eastman et al., 2017) on the predicted protein structure, allowing us to monitor estimated energy.

The next three sections examine how the information input in AF influences the results, providing insight into its predictive behavior. Sections 2.6 and 2.7 demonstrate the application of the VAIRO method to two distinct large macromolecular assemblies.

### 2.2 Proof‐of‐concept: predictions based on minimal information

As a baseline to understand how each information channel can steer predictions, we used VAIRO to run AF with minimal information by providing an empty features.pkl, thereby excluding both structural templates and MSA. In this setup, only the learned model parameters and the query sequence remain. These uninformed predictions provide a starting point to test how adding specific templates, modifying MSAs, or introducing sequence variants (modifications into the query sequence) can alter the predictions.

The small metamorphic protein Selecase (Lopez‐Pelegrin et al., 2014) is an extreme example of molecular plasticity, as three different conformations (five counting mutants) have been evidenced in crystals. Surprisingly, the uninformed prediction successfully reproduced one of these conformations, the monomeric structure (PDB 4QHF), with an root mean‐square deviation (RMSD) below 1 Å from the experimental structure (Figure 1a, Table S1). Supplying any of the experimental structures as template steers predictions toward its corresponding conformation (Figure 1b). Thus, the functional dimer structure (PDB 4QHH), in which a helix is remodeled into a beta‐hairpin, was reproduced only when its template was used. In this case, two best ranked predictions matched the 4QHH conformation while the remaining predictions matched the monomeric structure (4QHF). Providing all experimental structures simultaneously as templates led to the monomeric structure, so the 4QHH conformation was not represented despite the presence of its template. Similarly, a template truncated to polyalanine failed to produce the 4QHH conformation.

These results show that AF selects the conformation most strongly encoded by the available information and is driven toward more stable states (Table S1). Thus, the customization of this input can sample more conformational states.

With no recourse to database information, predictions typically fail as illustrated with the Rei‐T39K immunoglobulin domain (Usón et al., 1999), which reaches no successful fold solely from AF parameters. The predicted model shows how the all‐beta sandwich fold is disrupted by an alpha helix and the central disulfide bridge cannot be formed given the separation of the misplaced cysteines, yielding an RMSD of 3.8 Å over 79 Cα with the experimental structure 1BWW (Figure 1c). As expected, the default AF prediction easily succeeds and providing a complete correct template also reproduces the experimentally observed fold with an RMSD of 0.49 Å (Table S2).

The uninformed result yields a starting point to probe the effect of different external sources of information on the prediction (Figure 1d). To recover the correct fold from the uninformed prediction, we introduced mutations in the query sequence and informed local interactions with small templates or MSA (Figure 1d).

First, we introduced proline substitutions at residues 29–39 to discourage the spurious helix, since proline residues are known to disrupt helical geometry. Substitution in the first or last residues of the helix did not affect the prediction, whereas substitutions in central positions eliminated the helix and produced models much closer to the experimental structure, with RMSD below 2 Å and correct formation of the disulfide bridge.

Alternatively, we encouraged the formation of the disulfide bridge by providing partial templates or sequences. Using ALEPH (Sammito et al., 2013), we extracted a library of immunoglobulin domains containing 197 sequences corresponding to the seven and eight residues flanking the cysteines in the models. These fragments, encompassing 15 residues in total, represent the two β‐strands linked by the disulfide bond. Predictions informed by this MSA containing these sequences covering the partial templates, with or without the structural fragments as templates, improved the prediction compared to the uninformed run, correctly forming the disulfide bond and restoring the β‐sandwich fold. The overall deviation from the experimental structure was reduced, and per‐residue AF confidence predicted local distance difference test (pLDDT) increased, although the estimated ∆*G* did not improve significantly. The helix is still present and not disproven by its pLDDT, which remained higher than for the rest of the structure.

Combining the I31P mutation with the partial MSA, or equivalent templates gave the best result, with 0.79 Å RMSD from the experimental structure and an estimated ∆*G* below −2000 kcal/mol, approaching the value for the fully informed prediction (−2337 kcal/mol; Table S2). Using these improved models as polyalanine templates in subsequent prediction cycles with the original sequence yielded predictions even closer to the experimental structure. Importantly, mutations in the query sequence were reverted by using the resulting unrelaxed prediction as template for a subsequent prediction.

### 2.3 Proof‐of‐concept: template sequence dependency on predictions

**FIGURE 2:**
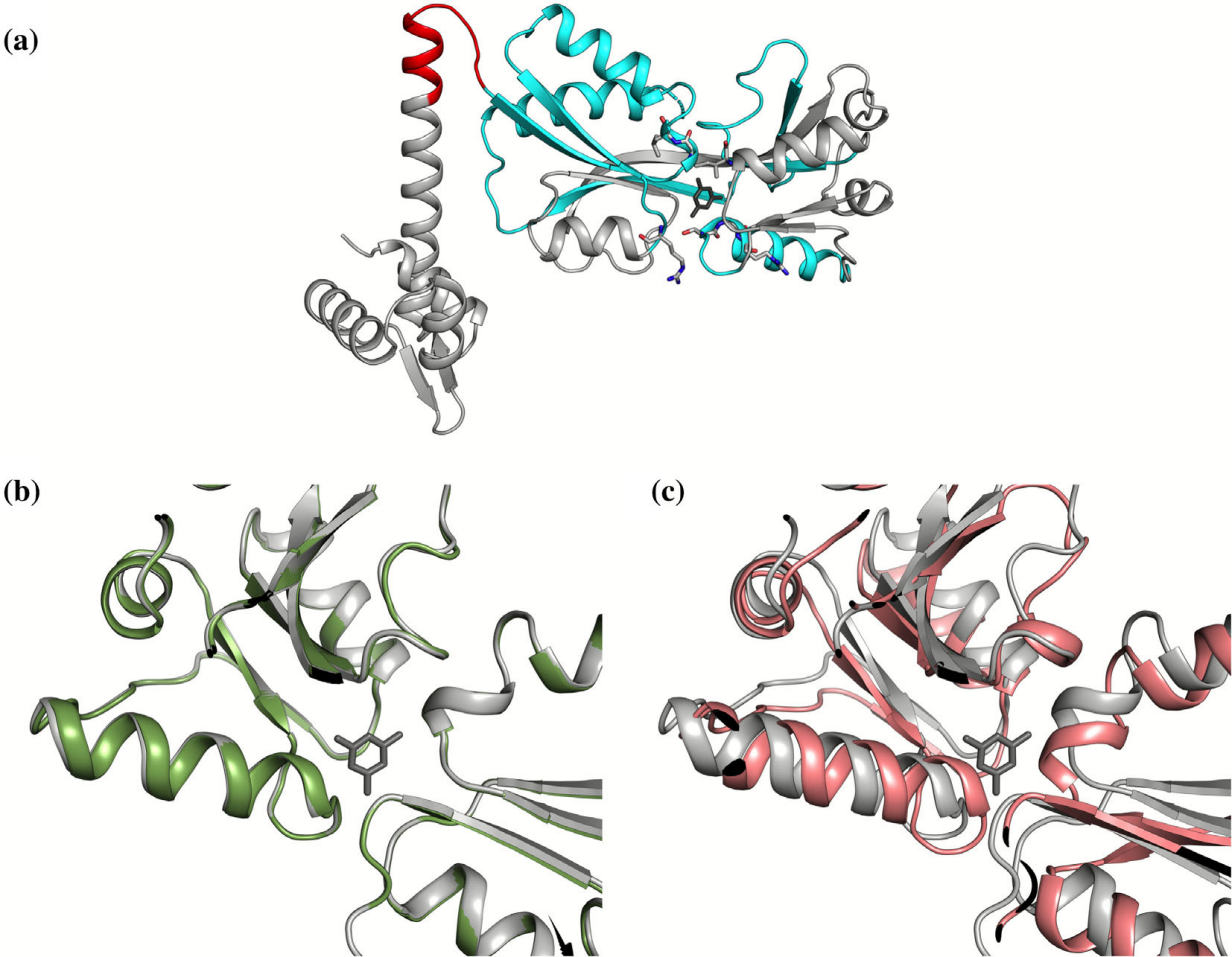
Differences in the prediction of AtzR in the environment of the effector binding site depending on the sequence of the template. (a) The AtzR template (PDB ID 7Z7J, chain A) in compact conformation in gray cartoon. The hinge region is shown in red, the secondary structure elements containing the seven residues binding the cyanurate effector (dark gray) are shown in cyan, the residues coordinating cyanurate are shown as sticks. (b) In green, prediction of the experimental AtzR with side‐chains in template; (c) in pink, prediction with polyalanine template.

We next examined how template modifications might induce a different weighting of specific regions that would influence AF predictions. As observed in the Selecase experiments, truncating a template to polyalanine changes its effect on the prediction. To explore this dependency with VAIRO, we generated templates with side‐chains truncated to the Cβ atoms but where the residue labels were changed to polyalanine or to match the query sequence (Castellví et al., 2022). For the 300 residues monomeric sequence of the Lys‐R type regulator AtzR (Porrua et al., 2007), a template annotated with the full AtzR sequence produced a model nearly identical to the experimental structure, characterized by a Cα RMSD of 0.35 Å, comparable to the one shown by independent copies of a single structure in a crystallographic determination. The largest overall variation is seen when the same template is annotated as polyalanine: neighboring regions come closer filling the void occupied by the cyanurate effector in the crystal structure, with an RMSD of 3.7 Å. Annotating only 10 residues in the hinge (81–91) with the AtzR sequence limits movement between domains and reduces the RMSD by almost half. Also, the environment of the ligand present in the experimental structure can be fastened setting the sequence of the ligand‐binding residues and their context. Details of the predictions to probe the effect of sequence identity between template and query sequence are summarized in Figure 2 and Table 1.

**TABLE 1:** Differences between prediction^a^ and template for AtzR monomer depending on template sequence.

Template	∆template cα RMSD (#aligned)	∆template cα (Cterm 92–300)	∆template cα (hinge 81–91)	∆template cα (ligand‐binding residues/SS)
AtzR with full sequence	0.35 (292)	0.31 (209)	0.37 (11)	0.37 (11)/0.23 (63)
AtzR polyalanine; seq at ligand‐binding SS^b^	1.78 (292)	1.36 (209)	0.76 (11)	0.76 (7)/1.00 (63)
AtzR polyalanine; seq at hinge (81–91)	2.09 (292)	1.81 (209)	0.62 (11)	0.62 (11)/1.82 (63)
AtzR polyalanine; seq at seven ligand‐binding res	3.39 (292)	1.42 (209)	1.78 (11)	0.63 (7)/1.11 (63)
AtzR polyalanine	3.69 (292)	0.86 (209)	1.8 (11)	1.8 (11)/1.9 (63)

^a^ Multiple sequence alignments containing AtzR sequence only; template monomer in bent conformation (PDB ID 7Z7J, chain A).

^b^ SS all residues in secondary structure region of the ligand‐binding amino acid.

### 2.4 Proof‐of‐concept: templates do not immobilize predictions

Building on the previous section, we next explored whether templates restrict or guide AF in predicting different conformations. The family of bacterial LysR‐type transcription regulators (LTTR) shows extremely different conformations in the experimental tetrameric structures so far described (Giannopoulou et al., 2021). We have guided AF using main chain templates of each of these experimental structures to test whether it would draw equivalent conformations for the sequence of AtzR. We have emulated classical homology‐modeling to interrogate whether corresponding states for the changes triggered by effector binding underlie all LTTR. The results show that templates influence but do not immobilize predictions. The predicted C‐terminal domain dimer invariably resembled more the yet undeposited experimental structure than the templates, showing that the prediction was not suppressed by the information provided. Considering the closest homologs, TsaR (Monferrer et al., 2010) (23% identity) renders one of the closest predictions to its template whereas the prediction with CbnR (Muraoka et al., 2003) (22% identity) differs essentially in the interfaces formed. In contrast, CbnR cannot be coaxed to adopt the conformation revealed in the AtzR structure (Figure S1). Beyond AF's pLDDT, dissociation energies estimated for the predicted multimers suggested which hypothetical conformations could be entertained and which were unlikely to exist in the predicted form (Castellví et al., 2022).

### 2.5 VAIRO description and implementation

The insight described in the proof‐of‐concept sections underlies the development of VAIRO. To explore a system, VAIRO can be configured to automatically identify possible structural states among the templates retrieved by AF. By default, the templates are clustered and AF is run using each cluster as a template set to guide predictions toward two structural states. Users can choose to subdivide templates into additional groups.

From this exploration or to address prior questions involving weak interactions and multiple conformations, VAIRO enables more directed customization, allowing prior knowledge to be integrated into the input provided to AF. Our implementation allows input modification at three levels (see Section 4.1):

1. Query sequence: Multiple query sequences can be provided when predicting oligomeric assemblies or protein–ligand complexes. All sequences are concatenated with linkers into a monomer for the prediction and disengaged into chains for the analysis. The user can modify (mutate) specific residues in the query sequence to favor or disfavor particular interactions or folds.
2. MSA: The user can choose to use the full MSA, restrict it to a subset of entries, or mask certain regions to emphasize the corresponding template.
3. Templates: The program provides full control over the templates supplied to AF. A user can specify a PDB ID, and VAIRO will automatically set it as a template for AF. The sequence of a template can be partially or completely modified—converted to polyalanine, substituted with another sequence, or replaced by the query sequence itself. Different templates can be assigned to different chains or regions in the prediction, reconstructing interactions. Additionally, users can supply a library of structural fragments or sequences containing specific interactions to guide AF toward desired conformations.

We recommend establishing a baseline for a given biological system, running VAIRO with defaults. The automatic analysis of input and results will help to fine‐tune the input toward a particular conformation. For further details and examples, see Structural analysis and clustering methods illustrated on the dynamics of the Sugar Transporter Protein 10 (STP10) family, and Figure S2.

### 2.6 Application to the architecture of the bacterial S‐layer

We next applied the VAIRO method to explore how guided predictions can reveal and assemble interactions within complex macromolecular systems. S‐layer proteins (SLP) are the building blocks of S‐layers, organizing into a self‐assembling array that covers the cell envelope of diverse archaea and bacteria (Buhlheller et al., 2024; Sagmeister et al., 2024). These structures essentially contribute to maintaining cell integrity, protecting against environmental stress, and facilitating interactions with host organisms (Fagan & Fairweather, 2014; Sleytr et al., 2014). Electron microscopy analysis of the two‐dimensional (2D) crystals formed by SLP from *Lactobacillus acidophilus*, SlpA, revealed the dimensions and planar symmetry of the S‐layer formed. SlpA encompasses three domains; the first two (D1, D2) form the self‐assembly layer facing the environment, while the C‐terminal domain (D3) is responsible for the attachment to the cell wall (Smit et al., 2001).

**FIGURE 3:**
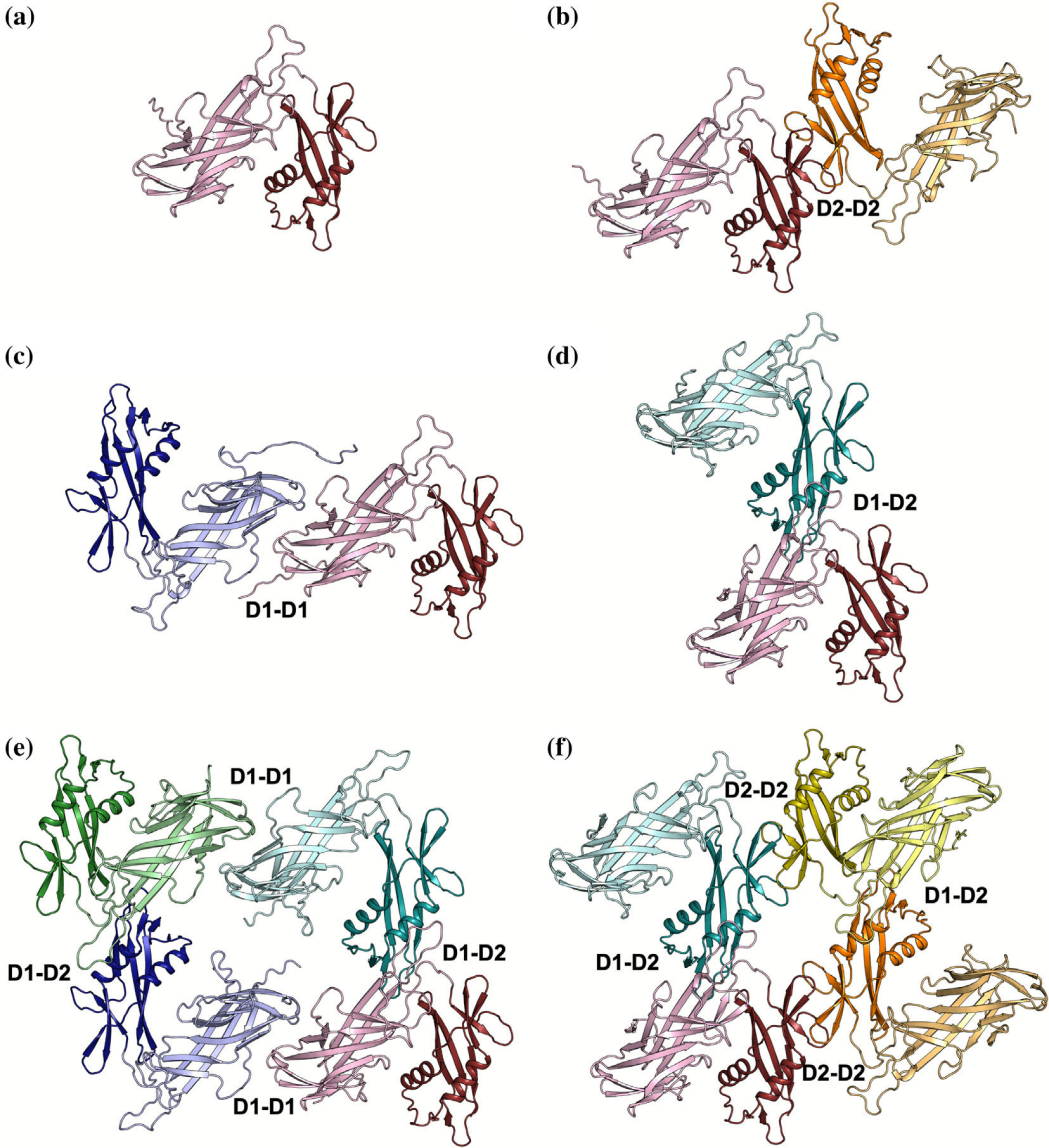
Application of VAIRO to the architecture of the *Lactobacillus acidophilus* S‐layer (SlpA); monomers are shown in different colors, with the N‐terminal D1 domain in a lighter shade than the following D2 domain. Interfaces are labeled with the domains involved. (a) Native AlphaFold (AF) prediction of the S‐layer proteins (SLP) monomer exhibiting only domains involved in the self‐assembly (D1 and D2). (b) SLP dimer associated through the D2 domains. (c) SLP dimer associated through the D1 domains. (d) SLP dimer associated through one D1 and one D2 domains. (e) SLP tetramer associated through D1 domains and through one D1 and one D2 domains. (f) SLP tetramer associated through D2 domains and through one D1 and one D2 domains. Figure S3 shows the pLDDT‐colored predictions.

The native AF prediction for the *L. acidophilus* SLP D1‐2 monomer (32–308) renders five equivalent models (Figure 3a) and a features.pkl array harvesting the information available in the databases regarding other known sequences and related templates. Experimental structures of the isolated D1 (7QLE) and D2 domains (8BT9) reveal interactions that would enable formation of a monodimensional chain. VAIRO can direct dimer prediction toward formation of the different interactions required for an extended 2D layer as summarized in Table S3. Addition of the template containing the D2 dimer seen in the 8BT9 structure truncated to polyalanine renders the dimer joined by this interaction (Figure 3b), estimated to be highly favorable by PISA (Krissinel, 2011). The corresponding prediction using the D1 dimer seen in the 7QLE crystal structure as polyalanine does not result in models displaying this interaction but rather the previous, more stable one. To retrieve the D1‐D1 interaction, it is necessary either to reduce the depth of the MSA or to mask the MSA in the region of the loops responsible for the D2‐D2 interaction (Figure 3c). Masking the most stable interaction allows additionally to see models displaying a third kind of favorable interaction, involving the first domain in one monomer and the second domain in the other (Figure 3d). Adding this D1‐D2 interaction through a template can be used to steer tetramer predictions joining D1‐bound dimers (Figure 3e), or alternatively joining D2‐bound dimers (Figure 3f). Presence of the full MSA—except for 19 residues masked, corresponding to the loops involved in the interface between D2 domains—allows to omit the monomer template. A hexameric tile, comprising all interactions in the SLP assembly part, is built on the same principle, selectively guiding predictions. As the sequence becomes too large for our hardware, it is run in mosaic mode, automatically decomposing and assembling on the two common domains the two tetramers described. In practice, the second assembly is seeded from the common domains from the best‐scored first prediction.

**FIGURE 4:**
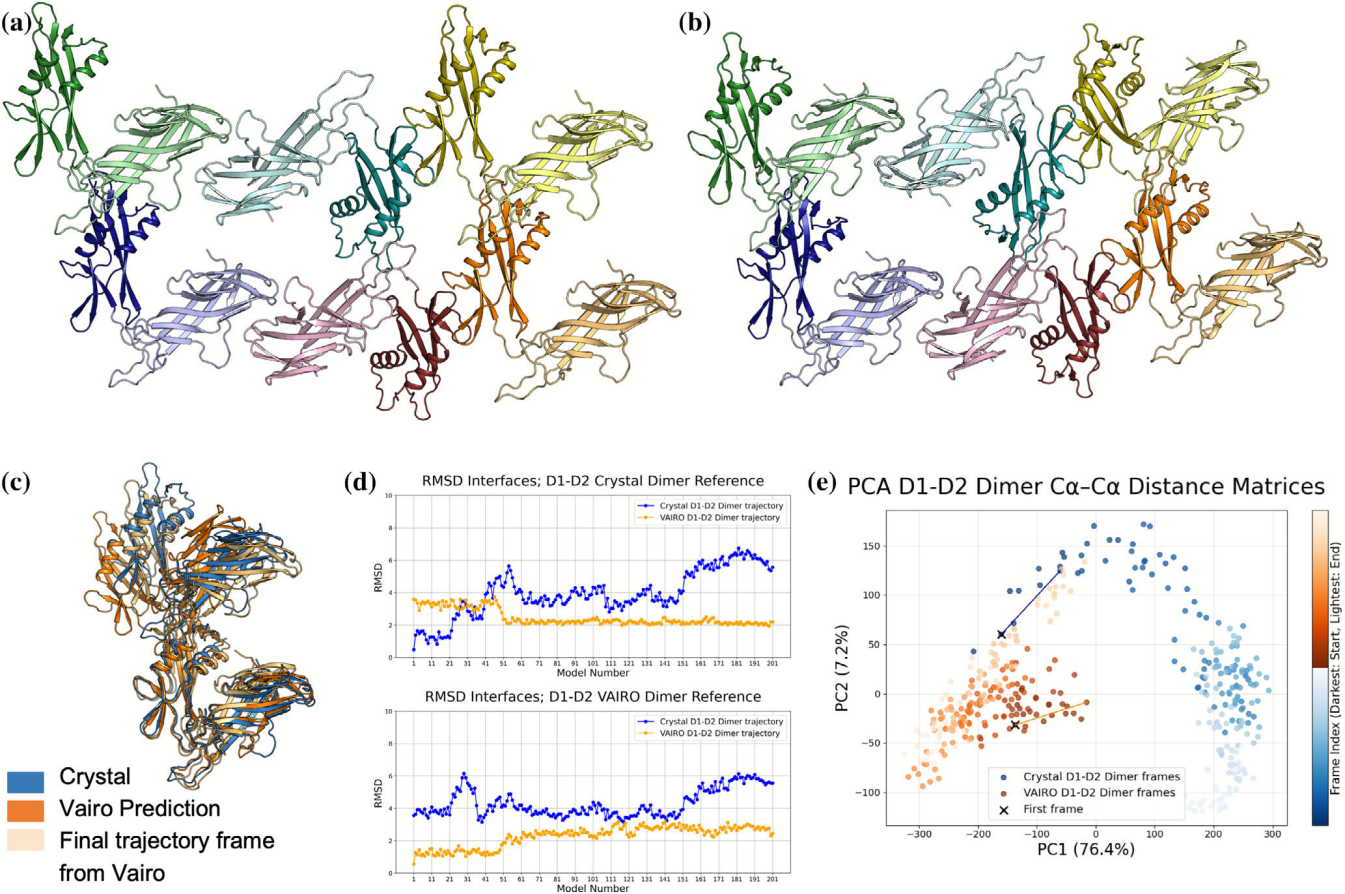
Static and dynamic comparison of experimental structure and prediction. (a) Hexamer in our crystallographic structure (9RPL). (b) Hexamer predicted by VAIRO. (c) Superposed D1‐D2 dimer from crystallographic structure (blue), prediction from VAIRO (orange) and the last frame in its molecular dynamics simulation (light orange). (d) RMSD comparison between the trajectory generated from the crystallographic structure in blue and the trajectory produced with the prediction from VAIRO in orange. The RMSD (calculated considering 74 residues corresponding to the interface D1‐D2) results from a subsample of 200 frames from each trajectory with the crystallographic structure (top) and the prediction from VAIRO (bottom) as reference. (e) Principal component analysis of the dimer crystal and prediction D1‐D2 trajectories as described in (d). Solid colors reflect the start of each trajectory and transparent colors the end. PC1 accounts for most of the variability in the data (76.4% of variance) while PC2 only the 7.2%. The plot reflects that both starting structures represent two different conformations. It also shows the trajectory from the prediction ends in the starting point of the trajectory coming from the crystal.

Subsequent determination of the crystallographic structure of *L. acidophilus* D1‐D2 SLP (Figure 4a) allowed validation of the model produced with VAIRO (Figure 4b). Overall, the predicted tetrameric or hexameric tiles are more curved than the surface in the crystal, mainly derived of a more skewed D1‐D2 association as seen in the RMSD calculated for the corresponding superpositions (5.93 Å, 514/514CA for D1‐D2 vs. D1‐D1 1.69 Å, 284/288CA and D2‐D2 1.46 Å, 214/215CA). Regarding higher associations, differences for the D1‐bound tetramer amount to 4.96 Å for 1040/1046, whereas the D2‐bound tetramer yields 7.66 Å for 1040/1046 atoms.

Rotation of the D1‐D2 interaction entails different atomic contacts between the experimental and model structures, mainly in the interactions between a loop in D1 and a helix in D2, as seen in the superposition shown in Figure 4c. To put these differences in a dynamic perspective: bacterial surface layers wrap a non‐planar bacteria and need to break and reform adapting to its life cycle, we performed molecular dynamics (MD) simulations. Figure 4c shows a movement of the prediction approaching the crystal structure, corresponding to a reduction in the RMSD for the interface residues versus the experimental reference (Figure 4d). Principal component analysis of the D1‐D2 trajectories shows the prediction approaching the experimental structure, which in turn ends with comparable differences to both references (Figure 4e). Furthermore, 100 ns simulations for experimental and predicted D1‐D1, D2‐D2, and D1‐D2 dimers were performed in the context of dimers, tetramers and hexamers, indicating a higher flexibility of the D1‐D2 interface (Figures S4 and S5).

### 2.7 Application to the Ami permease system from *Streptococcus pneumoniae*: clustering and direction from inward‐facing toward outward‐facing conformation

Next, we applied VAIRO to guide predictions toward different dynamic states in a permease system. The object of this study is an ATP‐binding cassette (ABC) transporter (Alcorlo et al., 2024; Durmort & Brown, 2015; Thomas & Tampe, 2020) composed of four subunits encompassing 1465 amino acids and a fifth periplasmic component out of several extracellular oligopeptide substrate binding proteins (SBP) of over 600 residues. In this system, the transmembrane heterodimer is formed by AmiC and AmiD, and two cytosolic ATPases, AmiE and AmiF, that power oligopeptide transport across the membrane through adenosine triphosphate (ATP) hydrolysis (Gilson et al., 1988).

We had experimental structures in peptide‐bound and peptide‐free states for several of the SBP but for all other components only homologous structures were available: few and distant for the membrane proteins and numerous, well‐conserved ones for the intracellular components. As a particularity, the role of both heterodimers corresponds to homodimers in some other systems. With this background, an AF prediction calculated through VAIRO in its mosaic mode in order to handle the large target yields a prediction, with energetically plausible interactions according to the energy estimates in PISA and favorable pLDDT with few exceptions limited to terminal residues and coils. The multimeric model presents an occluded channel and the structural traits mark a closed, inward‐facing conformation, with the transmembrane domains open to the cytoplasm. This conformation is equivalent to the one produced by either AF2 multimer with ColabFold or AlphaFold3, despite the ligand information (Figure S6).

The aim was to derive an alternative prediction of the complex representing its outward‐facing conformation to gain functional insight. Our previous tests to develop the method to automatically analyze and partition the information retrieved on seven transporter families are exemplified in Supporting Information S1 for the case of the STP10 family (Bavnhoj et al., 2021). In the case of the large Ami system, we started predicting the different interactions within the complex, guiding the alternative functional states. The complex can be divided into structural groups: the oligopeptide binding proteins (SBPs), the transmembrane dimer, and the ATPase cytoplasmic dimer.

**FIGURE 5:**
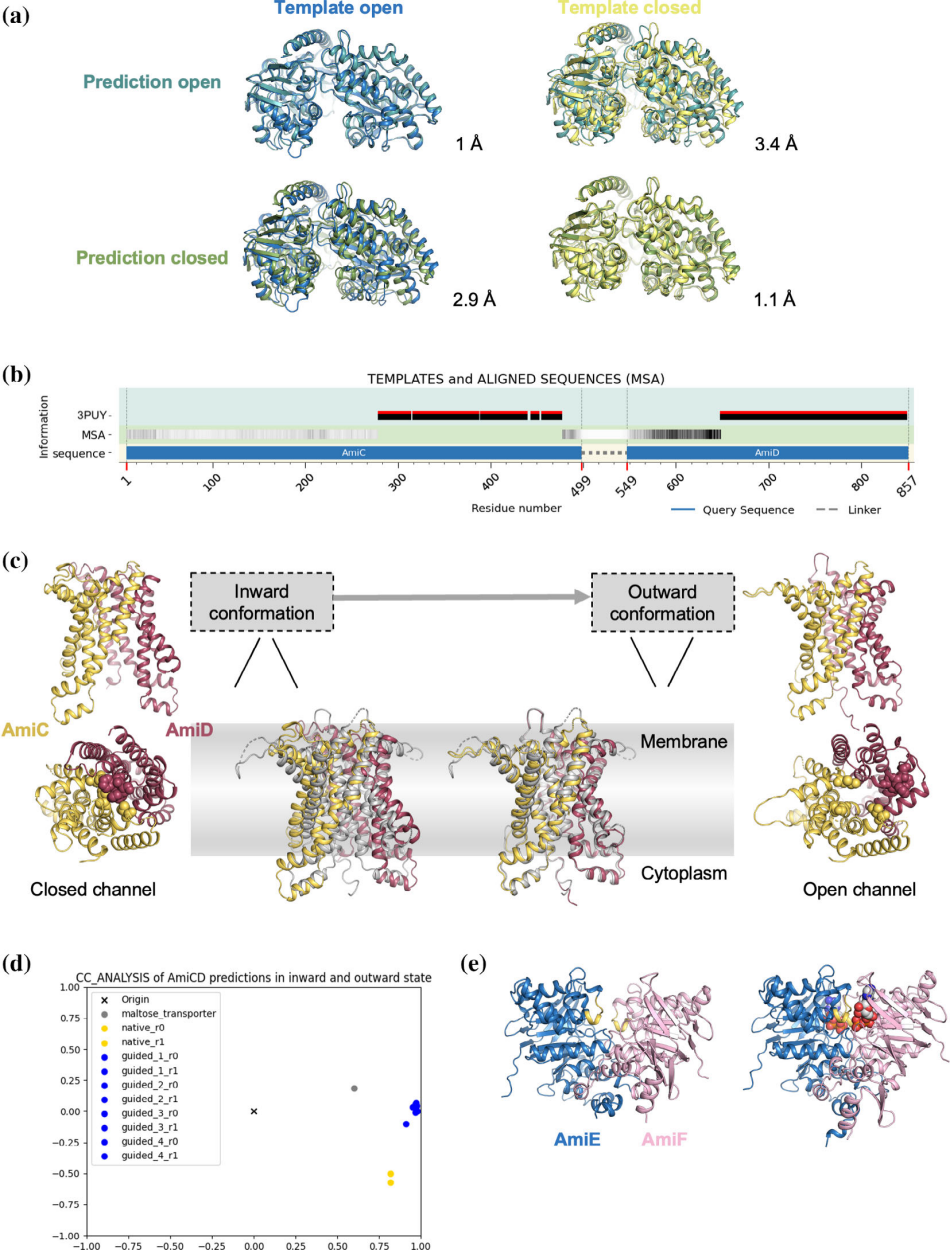
Template‐guided prediction of the components from the pneumococcal permease system in outward‐facing conformation with VAIRO. (a) AmiA predictions guided toward open (teal) and closed (green) states by the templates from the crystal structures of AliD open (blue) and closed (yellow), RMSD in Å for each of the superpositions involving 622 Cα; (b) Scheme of the information provided to AF replacing its native search. (c) Superpositions of the AmiC (yellow)‐AmiD (red) dimer, for the spontaneous inward conformation (left) and guided outward conformation (right) with the outward template 3PUY (gray); hydrophobic residues closing the entrance of the channel (inward conformation) or moving away to open the channel (outward conformation), are represented as spheres (d) CCANALYSIS of structural results showing discrimination of inward (yellow) and outward (blue) conformations. (e) AmiE‐AmiF dimer in both conformations, the ATP molecule is shown in the closed conformation. MSA, multiple sequence alignments.

Those SBP, for which we had no experimental structure or only in ligand‐free (open) or ligand‐bound (closed) state, were predicted with AF2 (Figure 5a). The availability of experimental structures for one SBP in both states allowed the classification of the resulting predictions as open or closed. The native AF prediction consistently yields the unbound state in open conformation. Closed conformations, with or without ligands in the recognition site, were guided using the experimental structures as templates. Also, our automatic identification of different structural groups among the templates retrieved by AF renders the respective open and closed conformation.

The transmembrane domain, formed by the heterodimer AmiC and AmiD, invariably renders an inward conformation in the native AF prediction. In the absence of experimental structures of these proteins, our integrated analysis of the templates used by AF, examining hinges between rigid groups and pairwise structural correlation with CCANALYSIS (Brehm & Diederichs, 2014; Diederichs, 2017) allowed us to discriminate two clusters representing the inward and the outward conformation. Most of the templates presented the inward conformation, corresponding to the resting state of the complex where the SBP is not bound. Three templates from a maltose ABC transporter contained the substrate arrested in the channel, displaying an outward conformation. The outward conformation is sustained by the energy delivered from the hydrolysis of ATP by AmiE and AmiF. The lower stability agrees with the lack of experimental structures in this particular conformation as well as the lack of native predictions reflecting this state. To guide predictions toward the outward conformation, the transmembrane region from one of the experimental templates representing this conformation corresponding to a crystal structure of a catalytic intermediate of the maltose transporter in a pre‐transitional state (3PUY; Oldham & Chen, 2011), with a sequence identity of 11%, was used as a template. The template was extended beyond the default sequence alignment relying on the secondary structure match to the inward prediction. Its sequence was changed to that of the pneumococcal transmembrane transporter and the sequence information retrieved in the native AF search was incorporated but masked at the template regions (Figure 5b). This yields models of the AmiCD dimer in the outward conformation (Figure 5c), as shown by CCANALYSIS (Figure 5d). Structural analysis reveals that the inward conformation is stabilized by a hydrophobic cluster of residues (phenylalanine residues in AmiD in a tight interaction with hydrophobic residues in AmiC) closing the upper region of the channel. This interaction can be shielded by mutating the residues involved in the query sequence but guidance with templates and MSA masking was found to be sufficient. The guided prediction shows this hydrophobic cluster receding away from the channel, which is thus opened (Figure 5c).

The ATPase heterodimer formed by AmiE and AmiF is also rendering an inactive conformation (ATP‐free state), lacking the contact between the signature motif LSGGQ (AmiE = YSGGM; AmiF = FSGGQ) in the helical subdomains and the P‐loop residues from the opposite domain (Figure 5e). To promote the active conformation (ATP‐bound), the same experimental structure of a maltose transporter (3PUY), used to predict AmiC and AmiD in outward conformation was used as template but limited to the region of the nucleotide‐binding domains.

**FIGURE 6:**
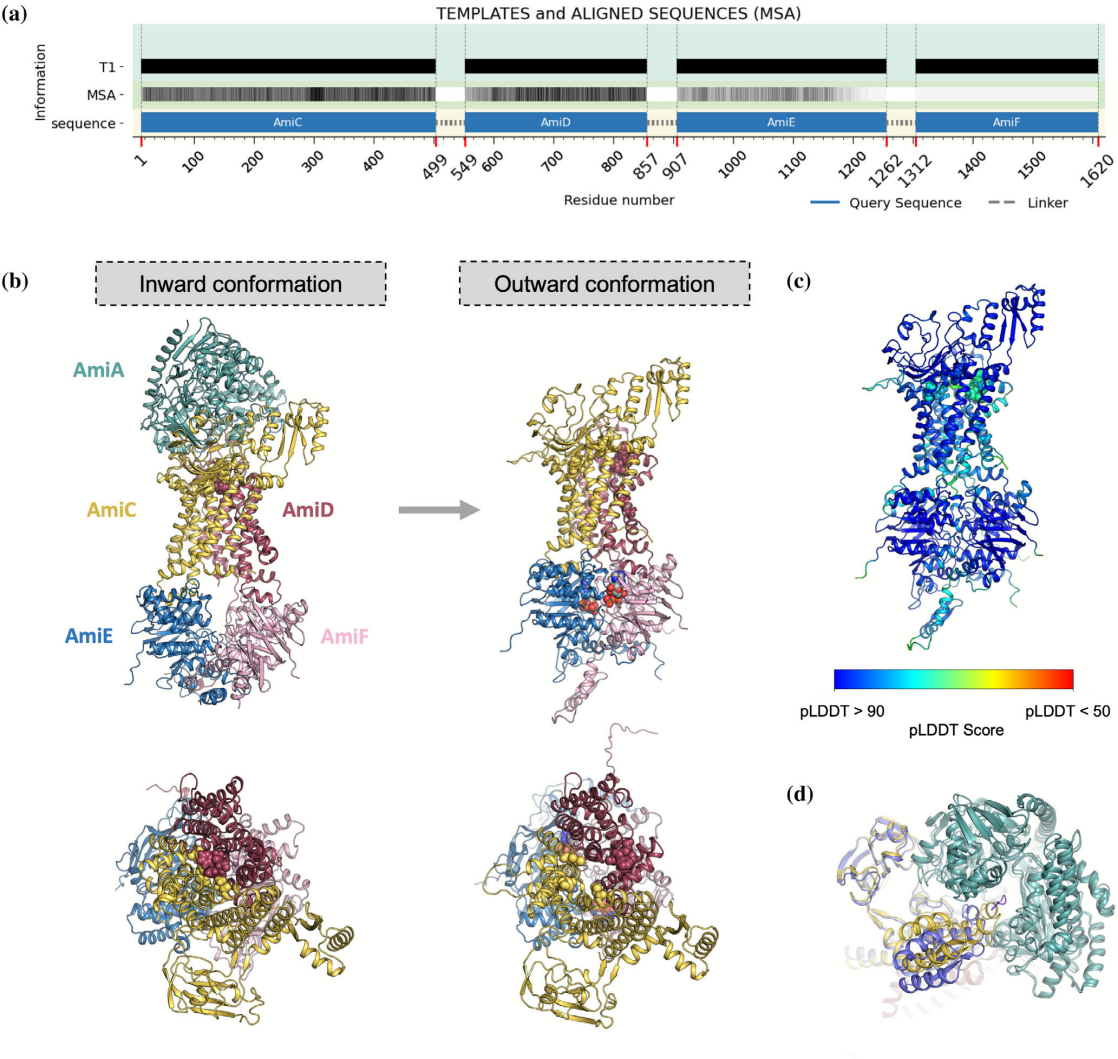
Prediction of the pneumococcal permease system with VAIRO. (a) Scheme of the information provided to AlphaFold replacing its native search to promote the outward‐facing conformation. T1 represents the template (black for same sequence), and the information from the MSA is represented in a black–white scale (darker for more sequences). (b) Side and top view of the complex (left) in inward conformation where the transmembrane channel is closed and the ATPase domain inactive; (right) in outward conformation with receding hydrophobic residues showing the aperture of the channel and the catalytic ATPase domain represented with its ATP ligand. (c) Outward conformation colored by pLDDT. (d) Top view detail for predictions in the interface between the SBP (teal), and AmiC (yellow) in the native prediction (open, absence of coordinated peptide) and AmiC (slate blue) with the experimental substrate‐bound AliD structure as template, the peptide is shown in purple. MSA, multiple sequence alignments.

To encourage the otherwise unreachable outward conformation, 3PUY was used as a template covering the complete ABC transporter. The template was previously aligned within VAIRO and lower homology regions were removed. Its sequence was changed to match the Ami permease transporter. The information supplied through MSA corresponds to that extracted from three different native features.pkl obtained with AlphaFold2 in native predictions of the trimer AmiC, AmiD, AmiE; the dimer AmiC, AmiD; and AmiF. The MSA of each features.pkl was masked in the region covered by the template. This yields a full complex prediction in outward conformation but renders modest pLDDTs (average 69). Using this prediction as a template for a subsequent iteration and increasing the information present in each MSA to cover all the structure while limiting it to the 30 top entries renders the outward prediction supported by high pLDDTs (average 88) covering the full structure shown in Figure 6a–c.

Regarding all components of the Ami permease system and interactions among them, our results indicate that the ATP‐bound state in AmiE and AmiF correlates with the outward conformation in the transporter (AmiC and AmiD), thus providing dynamic information on how the ATPase activity at the cytosol could promote oligopeptide transport from the periplasm into the cytosol of the cell. Figure 6d shows the native prediction of an SBP bound to AmiC and AmiD, in which a domain of AmiC is entering the peptide binding cavity of AmiA in its open conformation, whereas in our prediction, with the ligand‐bound conformation of AliD the loop is placed in a location that would allow for the peptide occupying the cavity as seen in the experimental structures.

## 3 DISCUSSION

Our results illustrate that the context of an AF prediction can be conveyed through information provided using three channels: aligned templates, MSA, and sequence variants. Surprisingly, for smaller proteins a successful prediction may result without templates or alignments, just from the learned parameters without the profiling step, as seen for the metamorphic protein Selecase. Subsequently, the addition of templates reveals alternative conformations. In most cases as for the immunoglobulin domain, uninformed predictions result in incorrect folds. From this baseline, predictions can be guided stimulating the elimination of an incorrect helix and the formation of the missing disulfide bond. Selecting or providing information to enforce prior knowledge opens a route to choose where to confer degrees of freedom in order to explore alternative states. The alternative information sources need to be balanced to have an effect.

In contrast to methods that rely on randomization of input data to explore multiple conformational states, VAIRO incorporates complementary information through deliberate modifications of the query and template sequences. We have found that the similarity between template and query sequence strongly conditions the weight with which the structural models influence the outcome. Identical sequence poses a strong restraint on the prediction, whereas providing a polyalanine structural model constitutes a weaker tie. In any case, templates do not immobilize predictions, which is not unexpected as the amino acid chemical drive for a preferred environment is implicit in the learned weights. This is observed in the case of the AtzR transcription regulator, which resembled more closely its own—undeposited—experimental determination than the tetrameric homologs provided as templates, preventing any further structure or sequence information. Local masking or reduction of sequence information contributes to enforce a template and may be needed to provide a view at a stage where an otherwise favorable interaction should not be formed.

Partitioning a native AF prediction into instances informed by consistent templates allows retrieving views into different dynamic states based on our prior knowledge. For the STP10 family of oligosaccharide transporters, we have drawn predictions targeted toward the inward and outward archetypal states (Figure S2). Automation and analysis of results are supported by the structural analysis of rigid fragments (Sammito et al., 2013) and their correlation, free energy obtained using OpenMM and dissociation energies estimated with PISA to assess the viability of the predictions and interfaces in the multimers.

We have implemented these conclusions in our program VAIRO and used it to bridge the scale spanning the SLP monomer structure prediction to the assembly of the corresponding bacterial S‐layer architecture and to access the predicted structure of the pneumococcal Ami permease complex in an activated state completing the dynamic view. The formation of S‐layers is a dynamic process, especially evident during the cell growth and division process, necessitating the rapid formation and breaking of bonds within the lattice (Comerci et al., 2019; Herrmann et al., 2020; Sleytr et al., 2014). Masking specific interactions in VAIRO allows to predict the less stable ones, which are fundamental in these dynamic processes, effecting the extended S‐layer. VAIRO also allows to predict the structure of the pneumococcal Ami permease system in outward‐facing conformation. AF prediction otherwise invariably leads to the more stable, inward‐facing conformation, closing a tight hydrophobic interface in the transmembrane channel.

These examples illustrate the broad applicability of VAIRO in detecting low‐affinity interactions, capturing them and providing representative predictions that are crucial to understand protein function.

Finally, it is important to consider VAIRO as a tool to extend classic homology modeling which depends on high‐sequence‐identity templates to reproduce known structural poses and interactions. Leveraging AF2's unique capabilities, VAIRO enables the use of low‐identity templates combined with MSA manipulations to generate dynamic conformational states and reveal transient interactions in a way that is not feasible with standard homology modeling techniques. VAIRO supports flexible input manipulation of the input information (adding specific templates, modifying MSAs or introducing sequence variants) to systematically explore alternative conformations.

## 4 MATERIALS AND METHODS

### 4.1 Implementation in our program VAIRO

**FIGURE 7:**
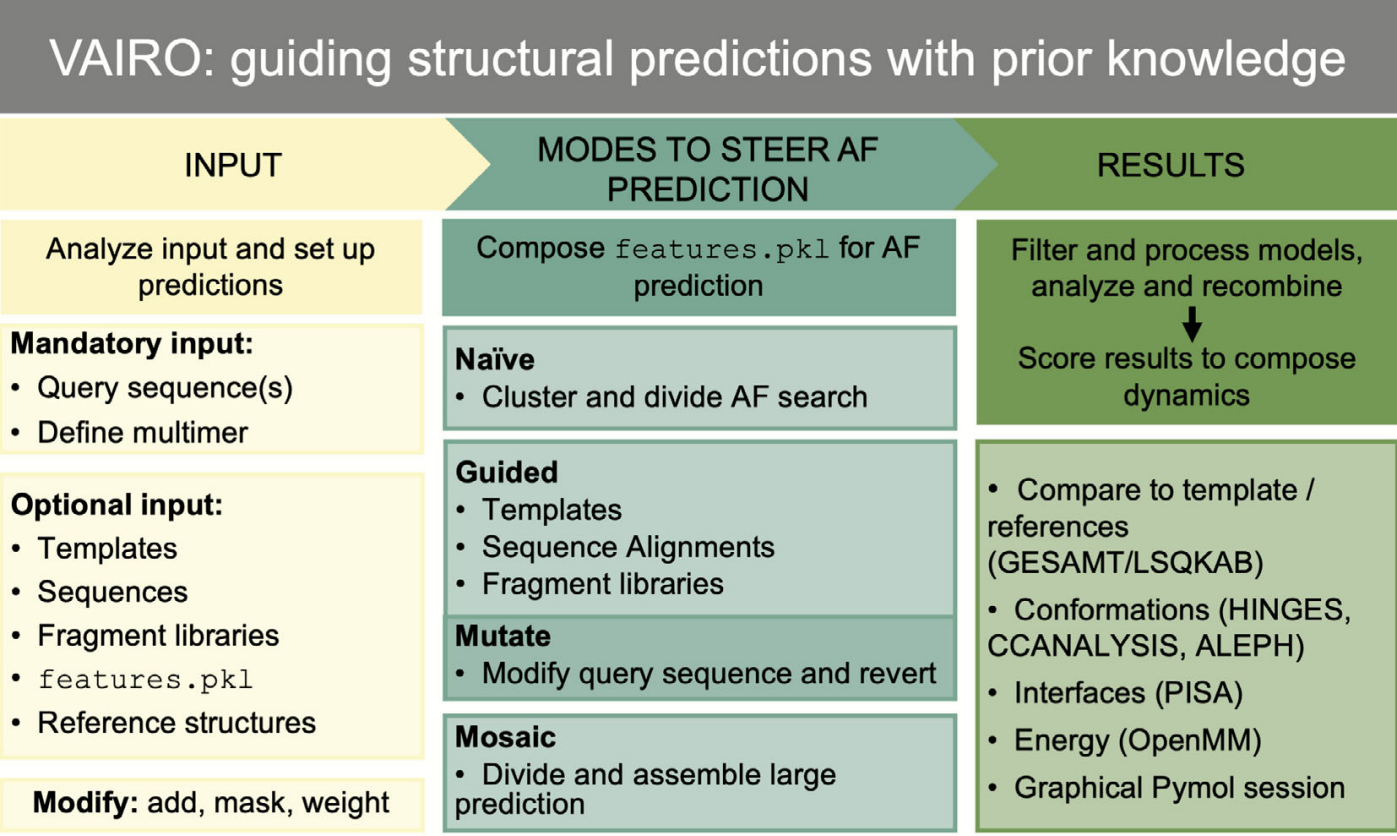
Scheme of VAIRO. The minimal input required consists of one or more sequences and their number of copies. Optionally, structural templates, sequences to integrate in the alignment or modify the templates, information gathered in a previous prediction for the same entity (a multidimensional numpy array saved in the file features.pkl of each run) may be input. Additional structures in PDB format may be given as references for comparison purposes in the analysis of results. Selections of how to modify the information using such items by addition, masking, or mutation can be specified or derived from the mode; the structure set up for prediction can be subject to four modes: The information selected by AlphaFold (AF) in a native run may be automatically analyzed and partitioned into different predictions targeting extreme states; otherwise, templates and/or multiple sequence alignments may be input to guide the prediction; the query sequence can be temporarily mutated; for large sequences, the prediction may be split and recomposed in the mosaic mode. Results are evaluated in terms of stereochemistry, structure, interactions, and energy.

VAIRO offers a range of methods to modify or select the information provided to AF in order to condition the predictions. This aims to explore and cover a range of interactions and conformations, which would otherwise be elusive when representing comparatively less stabilized states. As illustrated in Figure 7, different modes can steer the prediction: the main mode guides predictions with experimental data by using templates directly to introduce prior knowledge on a given conformational or active state. Concomitantly, the aligned sequence information (MSA) may be input, modified, lessened, or locally masked. Alternatively, the naïve mode halts a default prediction run after its native search for templates and related sequences, automatically groups the selected templates, and subdivides the prediction process to independently follow structurally consistent directions. Along with these two main modes, other convenient operations are facilitated. The query sequence can be mutated to discourage structural interactions absent in a functional state. This triggers a two‐step process as the mutation is reverted in a subsequent prediction with the previous result as template. Finally, the mosaic mode is provided to allow subdivision of large structures into overlapping predictions, process these individually, and lastly merge them to make larger sequences and complexes amenable to modest resources. VAIRO clusters the resulting models and identifies the best prediction considering pLDDT score, structural soundness (Ramachandran outliers and compactness) and consistency with the information provided (presence of interactions, RMSD to templates). The potential energy of each model is assessed to identify the most energetically favorable conformation. Protein–protein interfaces are classified according to their surface area, estimated Δ*G*, and number of hydrogen bonds. VAIRO generates structural superpositions of all predictions, templates, and reference experimental structures—if provided by the user—to assess the extent of structural divergence. This information is provided in the html output and through a comprehensive PyMOL session to facilitate visualization and comparative analysis of all predictions. Programs used are GESAMT (Krissinel, 2012), LSQKAB (Kabsch, 1976), OpenMM (Eastman et al., 2017), PISA (Krissinel, 2011), available through the CCP4 suite (Agirre et al., 2023); HHsearch (Steinegger et al., 2019), distributed with AF; and ALEPH (Medina et al., 2019), HINGES, and CCANALYSIS (Brehm & Diederichs, 2014; Diederichs, 2017) developed for VAIRO.

The structural analysis required for automation and interpretation can be linked to the common frame of the sequence to be predicted. In AF, information can only be input matched to individual amino acids at specific positions in this sequence. To exploit this intrinsic alignment property, we developed HINGES: rigid group decomposition and RMSD calculation to establish the range of structural variability. Then, with PDB2CC‐CCANALYSIS, a structural correlation coefficient is calculated for each pair of overlapping structures and from these data, multidimensional scaling optimizes relative positioning of the structures projected into a lower‐dimensional space. The procedure is agnostic to the actual data, but differences reflect the nature of the sample set. For instance, static changes within a population encompassing different families can be separated, or dynamic changes are reflected, positioning structures along the path of conformational movements (Figure 5c). In either case, random and systematic differences are discriminated by CCANALYSIS, and clustering can focus on the significant groups. Finally, the general landscape is related to the most significant local differences in terms of structural units, identified with ALEPH. For an illustration, see in Supporting Information S1 its application to the dynamics of the Sugar Transporter Protein 10 (STP10) family. Methods are described in technical detail in Supporting Information S1.

VAIRO is distributed via PyPI and the code is also available on Github (https://github.com/arcimboldo-team/vairo). We provide an installer that verifies the proper configuration of required external programs (e.g., MAXIT, PISA, and CUDA). Additionally, it sets up a Conda environment with all the required Python packages, including VAIRO and AlphaFold2 (v2.2.4 was used in the cases described). Furthermore, data are on our website (http://chango.ibmb.csic.es/VAIRO). All operations described in this work are supported in the distributed version and have been automated as far as possible, while allowing user control to override defaults. A graphical user interface is available.

### 4.2 SLP protein production and structure determination

The protein construct SlpA_ac_I‐II ΔN17 was cloned, expressed and purified as described in detail in a previous publication (Sagmeister et al., 2024). Restriction cloning with *Nco*I and *Xho*I into the vector pET28a(+) was performed with the forward primer 5′‐TATATCCATGGGCAAGTACGATGTTGATGTAA‐3′ and reverse primer 5′‐TATATCTCGAGATTAGGAACAGTAACAACTACT‐3′, using SlpA_ac_FL as template. Expression was performed in *Escherichia coli* BL21‐CodonPlus (DE3) RIL (Agilent Technologies) at 20°C overnight by induction with 0.5 mM isopropyl β‐D‐1‐thiogalactopyranoside. Protein was purified by Ni‐IMAC using the lysis buffer (25 mM imidazol, 50 mM N‐2‐hydroxyethylpiperazine‐N‐2‐ethanesulfonic acid (HEPES) pH 7.5, 300 mM NaCl), the same for loading and washing the column and an increased concentration of 250 mM imidazol for elution. Size exclusion chromatography was performed on an ÄKTA pure chromatography system (Cytiva, Sweden) with a Superdex 200 Increase 10/300 GL column with the buffer (25 mM HEPES pH 7.5, 150 mM NaCl).

Monodisperse, dimeric protein was concentrated to 4 g/L. Initial crystallization screening was performed with the commercial screens JCSG+ Eco and ShotGun (Molecular Dimensions). Optimization of crystallization yielded the final condition of 20% polyethylene glycol 3350, 50 mM sodium formate and 150 mM trisodiumcitrate and a drop ratio of 0.3 μL of condition mixed with 0.3 μL of protein solution in Swissci 3 lens vapor diffusion crystallization plates containing 35 μL screen condition in the reservoir and incubation at 20°C.

Crystals were frozen in liquid nitrogen without further cryoprotection and sent for data collection at ID30A‐3 at the ESRF (Grenoble, France). Data processing was performed with XDS (Kabsch, 2010), merging and anisotropic resolution cutoff was performed with STARANISO (Tickle et al., 2018). The structure was solved by molecular replacement in Phaser (McCoy et al., 2007) using the search models 7QLE and 8BT9. Automated refinement was performed using REFMAC5 (Murshudov et al., 2011) and phenix.refine (Afonine et al., 2012), and manual model building in Coot (Emsley & Cowtan, 2004). The final model and structure factors were deposited at the PDB with the accession code 9RPL. Data collection and refinement statistics are summarized in Table 2.

**TABLE 2:** Data collection and refinement statistics.

9RPL		
Data collection		
Wavelength (Å)	0.96770	
Resolution range (Å)	68.125–1.929 (2.164–1.929)	
Space group	C 2	
Unit cell (*a b c* [Å], *α β γ* [°])	149.1 50.6 118.6	90 113.9 90
Total reflections	132,050 (6770)	
Unique reflections	37,108 (1855)	
Multiplicity	3.6 (3.6)	
Completeness (%)	92.1 (60.7)	
Mean *I*/sigma (*I*)	8.3 (1.8)	
Wilson *B*‐factor (Å^2^)	31.61	
*R*‐merge	0.095 (0.723)	
*R*‐meas	0.113 (0.851)	
*R*‐pim	0.059 (0.4444)	
CC1/2	0.99 (0.697)	
Refinement		
Reflections used in refinement	37,103 (277)	
Reflections used for *R*‐free	1838 (13)	
*R*‐work	0.2042 (0.3439)	
*R*‐free	0.2432 (0.7789)	
Number of non‐hydrogen atoms	4074	
Macromolecules	3793	
Ligands	21	
Solvent	260	
Protein residues	523	
RMS (bonds) (Å)	0.004	
RMS (angles) (°)	0.63	
Ramachandran favored (%)	98.07	
Ramachandran allowed (%)	1.93	
Ramachandran outliers (%)	0.00	
Rotamer outliers (%)	0.48	
Clashscore	1.98	
Average *B*‐factor (Å^2^)	35.92	
Macromolecules	35.86	
Ligands	44.29	
Solvent	36.15	

*Note*: Statistics for the highest‐resolution shell are shown in parentheses.

### 4.3 Molecular dynamics simulations

MD simulations were performed with GROMACS, version 2024.2 (Hess et al., 2008) compiled in single precision, using OPLS‐AA/L all‐atom force field (Kaminski et al., 2001). Starting structures are derived from the x‐ray surface layer protein of *L. acidophilus* and from its prediction with VAIRO. We performed three replicas of 100 ns MD simulations (see Supporting Information S1).

## AUTHOR CONTRIBUTIONS

**Josep Triviño:** Validation; methodology; software; investigation. **Elisabet Jiménez:** Methodology; validation; investigation; data curation. **Christoph Grininger:** Investigation; resources. **Iracema Caballero:** Writing – original draft; writing – review and editing; visualization; investigation. **Ana Medina:** Conceptualization. **Albert Castellví:** Conceptualization. **Giovanna Petrillo:** Resources. **Fernando Govantes:** Resources. **Theo Sagmeister:** Resources. **Martín Alcorlo:** Resources. **Juan A. Hermoso:** Resources. **Massimo D. Sammito:** Conceptualization. **Kay Diederichs:** Methodology; software. **Tea Pavkov‐Keller:** Resources; investigation; supervision. **Isabel Usón:** Investigation; writing – original draft; writing – review and editing; supervision; methodology; software; conceptualization.

## Supporting information


**Data S1.** Supporting Information.

## Data Availability

The data that support the findings of this study are openly available in the Protein Data Bank at https://www.rcsb.org/, reference number 9RPL.
