# Supplementary material for: Guiding AlphaFold predictions with experimental knowledge to inform dynamics and interactions with VAIRO
Source: Protein Sci. 2026 Jan 24;35(2):e70481. doi: 10.1002/pro.70481 (PMC12831285; doi:10.1002/pro.70481)
Supplement: Supplementary file 1 — Data S1. Supporting Information. [file PRO-35-e70481-s001.docx]

**SUPPLEMENTARY INFORMATION**

**Table S1.** Comparison between experimental structures of *Methanocaldococcus jannaschii* Selecase and predictions depending on the external information. (§ Predictions based on minimal information)

| Information templates | ∆ 4QHF Cα rmsd (#aligned) | ∆ 4QHH Cα rmsd (#aligned) | ∆ 4QHI_B Cα rmsd (#aligned) | Energy Kcal/mol |
| --- | --- | --- | --- | --- |
| Bare sequence | 0.96 (106) | 1.42 (78) | 1.23( 94) | -1543.52 |
| 4QHF | 0.36 (108) | 1.35 (76) | 1.26 (94) | -1549.37 |
| 4QHF polyalanine | 0.85 (106) | 1.43 (77) | 1.3 (93) | -1656.47 |
| 4QHH | 1.22 (77) | 0.55 (108) | 0.85 (80) | -1169.23 |
| 4QHH polyalanine | 0.92 (106) | 1.28 (76) | 1.3 (93) | -1661.73 |
| 4QHI_B | 1.21 (94) | 0.94 (78) | 0.38 (109) | -1478.53 |
| 4QHI_B polyalanine | 0.94 (106) | 1.41 (77) | 1.3 (93) | -1649.09 |
| All templates | 0.58 (108) | 1.19 (76) | 1.03 (94) | -1577.01 |
| All templates polyalanine | 0.96 (106) | 1.4 (77) | 1.29 (93) | -1578.98 |
| Default AF | 0.67 (108) | 1.35 (78) | 1.32 (95) | -1696.65 |

**Table S2.** Characterization of predictions with minimal information for the immunoglobulin domain sequence ReiT39K corresponding to the crystal structure 1BWW. (§ Predictions based on minimal information)

| ReiT39K | Template, changes | MSA | Mutations | Difference to 1BWW Cα rmsd / core^a^ | %α / %β | pLDDT | Energy Kcal/mol |
| --- | --- | --- | --- | --- | --- | --- | --- |
| Minimal | – | – | – | 3.77 / 79 | 15 / 52 | 44 | -1498 |
| Mutation | – | – | 30 PRO | 3.33 / 91 | 0 / 63 | 47 | -1641 |
| Best Mutation | – | – | 31 PRO | 1.97/ 89 | 0 / 65 | 66 | -2030 |
| Revert best Mutation | – | – | 31 PRO | 1.93/ 91 | 0 / 70 | 76 | -2163 |
| MSA | – | +SSLIB | – | 2.82 / 92 | 8 / 54 | 55 | -1422 |
| Templates | +SSLIB | – | – | 2.69 / 89 | 12 / 56 | 45 | -1516 |
| MSA+ templates | +SSLIB | +SSLIB | – | 2.58 / 91 | 9 / 54 | 55 | -1409 |
| Mutation+ MSA | – | +SSLIB | 31 PRO | 1.74/ 104 | 0 / 65 | 65 | -1897 |
| Mutation+  Templates | +SSLIB |  | 31 PRO | 1.92/88 | 0 / 65 | 61 | -2007 |
| Mutation+ MSA+  templates | +SSLIB | +SSLIB | 31 PRO | 1.51/97 | 0 / 67 | 68 | -1890 |
| Revert Mutation+ MSA | – | +SSLIB | 31 PRO | 0.95/ 98 | 0 / 81 | 87 | -2236 |
| Revert Mutation+  Templates | +SSLIB |  | 31 PRO | 1.5/93 | 0 / 78 | 89 | -2183 |
| Revert Mutation+ MSA+  templates | +SSLIB | +SSLIB | 31 PRO | 0.79/89 | 0 / 76 | 84 | -2188 |
| Default AF | default | default | – | 0.49 / 109 | 0 / 74 | 98 | -2337 |
| Identical | 1BWW | – | – | 0.27 / 109 | 0 / 72 | 98 | -2439 |
| Mainchain | 1BWW, ALA | – | – | 0.60 / 109 | 0 / 74 | 94 | -2372 |
| Cut | 1BWW 18-42 | – | – | 1.08 / 95 | 0 / 68 | 83 | -2165 |
| Cut -ALA | 1BWW 18-42 ALA | – | – | 3.48 / 63 | 9 / 50 | 43 | -1381 |

**^a^**The number of core residues superposed best characterizes if structures are equivalent**.**

SSLIB is a library of random immunoglobulin structure fragments or their corresponding sequences comprising 20 distinct representatives, each encompassing 15 residues, forming two β-strands linked by a disulfide bond.


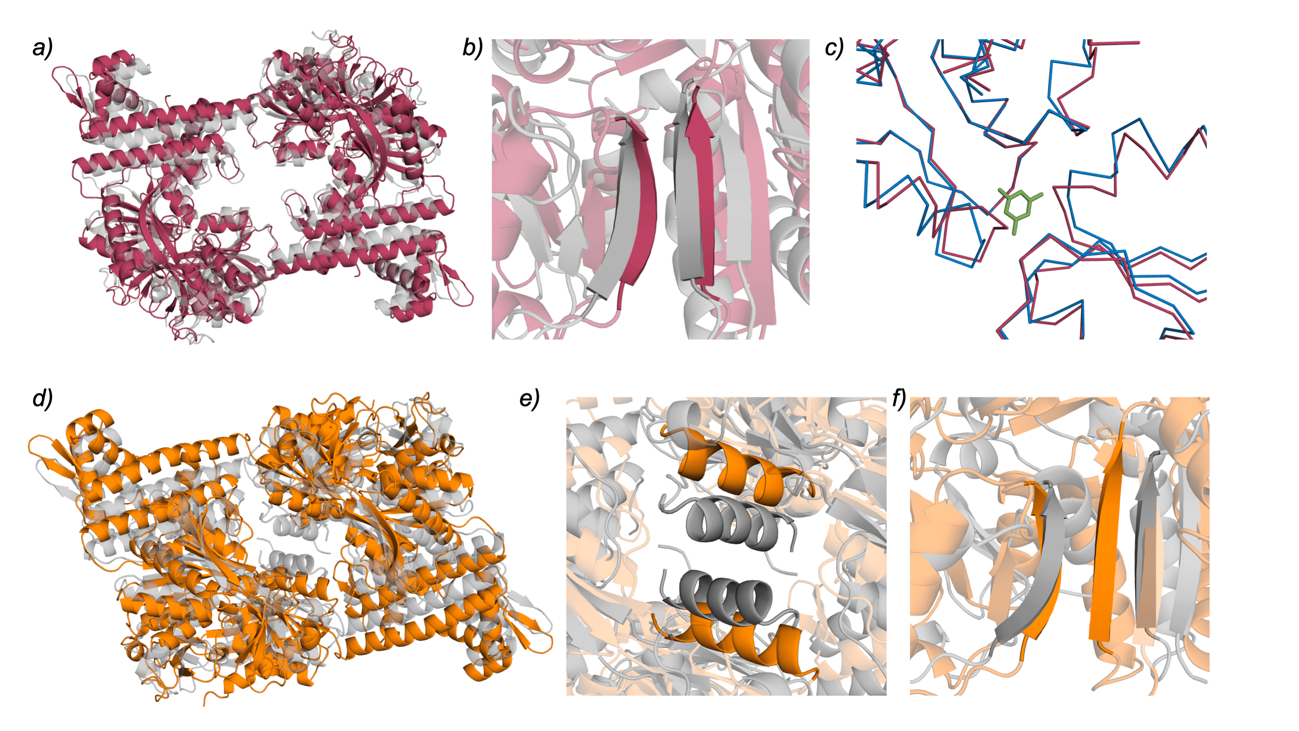


**Figure S1.** a) Superposition of the predicted AtzR model (magenta) after TsaR (3FXQ) template (grey). b) Dimerization of the C-terminal domains for prediction and template (grey). c) Detail of the ligand-binding area in the AtzR experimental structure (blue). In green, ligand found in experimental AtzR. d) Superposition of CbnR (1IXC) used as template for the prediction (grey) and the resulting AtzR model (orange), e) changes in the central interface closing the tetrameric ring. f) changes in the beta-strands from the dimerization interface of the effector binding domains. Note that the secondary structure elements highlighted in e) and f) correspond to residues aligned in AtzR and CbnR.

**Structural analysis and clustering methods illustrated on the dynamics of the Sugar Transporter Protein 10 (STP10) family**

The analysis of the structural landscape required to steer predictions automatically and analyze results can be linked to the common frame of the sequence to be predicted. In AF predictions, information can only be input by matching individual amino acids to specific positions in this sequence. To make use of this intrinsic alignment property, we developed ancillary methods, the first program, HINGES, uses rigid group decomposition and rmsd calculation to establish the range of structural variability. Then, with PDB2CC-CCANALYSIS, a structural correlation coefficient is calculated for each pair of overlapping structures and from these data, multidimensional scaling optimizes relative positioning of the structures. The differences may arise from diverse causes depending on the nature of the sample set. For instance, static changes within a population encompassing different families can be separated; or dynamic changes are reflected, positioning structures along the path of conformational movements. In either case, random and systematic differences are discriminated by CCANALYSIS and clustering can focus on the significant groups. Finally, the general landscape is related to the most significant differences in terms of structural units, identified with ALEPH.

Limiting the depth of the MSA used by AF was seen to diversify the conformations retrieved in the case of seven families of transporters. In contrast, native predictions would tend to render models reflecting a single state. We used these transporter families and the experimental structures identified as representatives for the inward and outward-facing states and selectively drew the corresponding conformations.

In the case of STP10 from *A*. *thaliana*, experimentally determined structures for that sequence are available in complex with glucose in the outward occluded state (6H7D (Bavnhoj, Paulsen, Flores-Canales, Schiott, & Pedersen, 2021)) and 7AAR and 7AAQ represent the inward and the outward open conformation respectively (Figure S1). Predictions using each of these templates and the MSA retrieved by AF in its native prediction reproduce the respective conformations, as evidenced by the rmsd of 0.6 Å for the inward conformation versus 7AAR and 0.2 Å for the outward conformation versus 7AAQ. Beyond this baseline, the interest of guiding the prediction would be to draw selectively the different states in related structures lacking an experimental determination.

For the STP10 transporter sequence a native AF prediction identifies 20 templates of comparable coverage and mutual overlap. The sequence identity between each of these templates and the query differentiates two groups, of ~30%, and <15%, respectively. Our program HINGES identifies the residues at which two structures need to be split for the resulting rigid groups to produce the lowest differences. It also differentiates two groups, within each of which pairwise Cα rmsd lie below 7 Å, in the range of secondary structure reorientation. In contrast, differences above 10 Å separate templates from very different identity. Further, it is seen that the main differences among both groups can be traced to the residues spanning 237 to 279 and that within 3 hinge-partitions the total rmsd drops below 5 Å for all pairs and structures become similar enough to represent comparable architecture in their folds.

Our PDB2CC-CCANALYSIS (Figure S1a) shows the results of the bidimensional clustering, where differences are reflected in the relative angles and confidence in the distance to the origin. Based on this analysis, VAIRO decouples both groups of templates and generates the input for two separate predictions (included in the *a posteriori* analysis). The plot shows a clear separation between the group of carbohydrate transporters and a group of proton-coupled, peptide symporters. Furthermore, the entries in the STP10 family are subdivided and sorted with inward conformations at the top leading to outward at the bottom (Figure S1b). The predictions for the two clusters also reflect their position at the extremes of the sugar transporters in the plot. This illustrates that CCANALYSIS will separate populations but that the nature of the differences cannot be taken for granted. The low sequence identity and large rmsd are used to filter out the group of peptide transporters, and the template of identical sequence 6H7D is also removed. The remaining templates on their own give a clustering consistent with the previous one, from which the extreme PDBs are selected to guide two separate predictions (Figure S1c). Given their overall low identity, their sequences are set to the target sequence to increase their weight and the natively retrieved MSA is reduced from 20 to 5 lines. The resulting rmsd lie 1.26 Å from inward conformation (7AAR) and 1.29 Å from outward conformation (7AAQ), respectively (Figure S1c). A further analysis step to connect the overall changes abstracted in CCANALYSIS with the differences in local structure is performed with ALEPH. Figure S1d highlights the largest differences found in angles of secondary structure elements for the outward prediction against the inward prediction as reference.


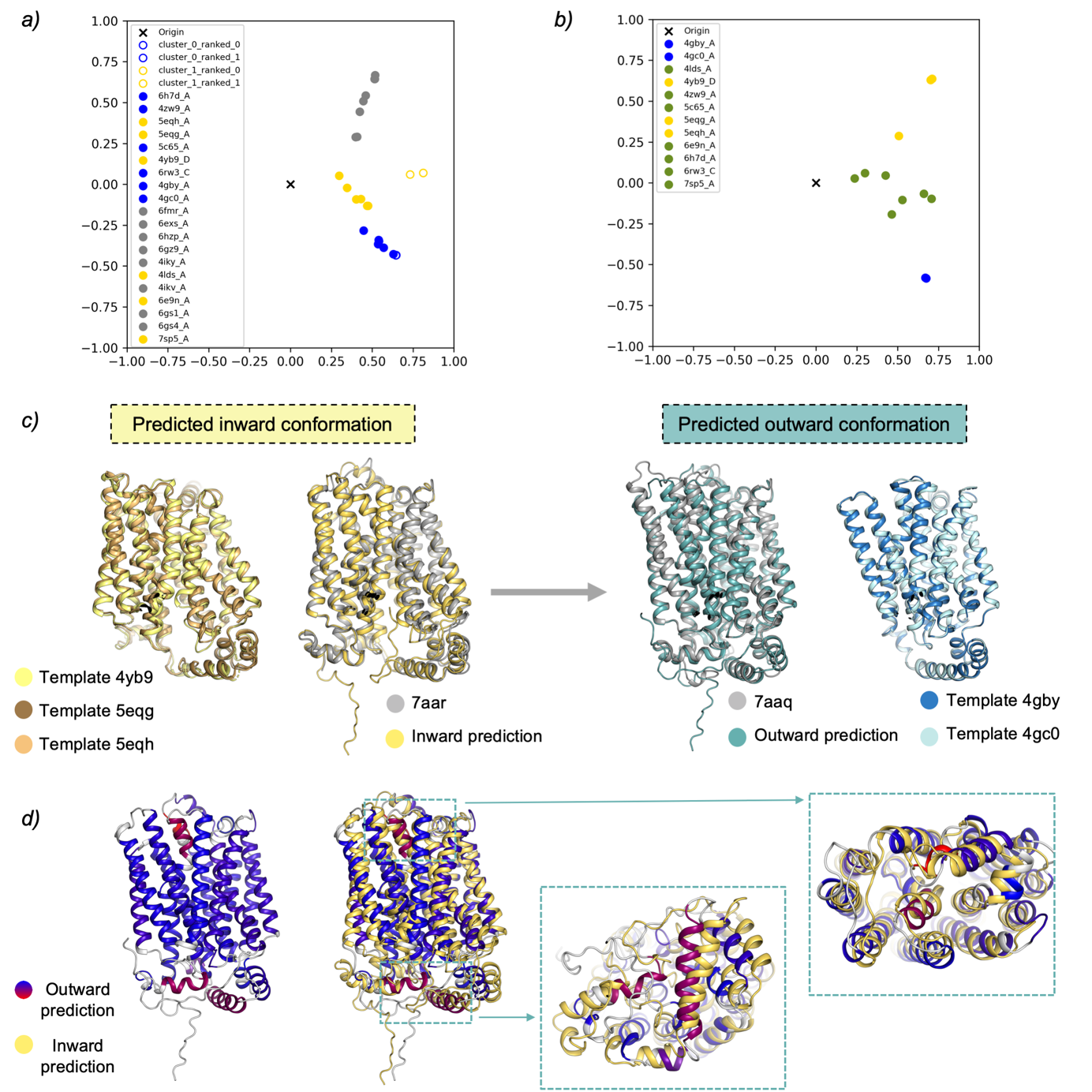


**Figure S2.** Transporter clustering and direction towards inward open and outward open states. a) Automatic assessment of all templates selected in a native AF run with CCANALYSIS separates peptide from sugar transporters and sorts the latter by their position according to the inward / outward dynamic state. b) Filtering out distantly related structures allows to identify two groups of experimental structures of homologs clearly representing the inward (4YB9, 5EGQ and 5EQH) and outward (4GBY and 4GC0) facing conformations, which are used as templates to guide separate predictions. c) Predictions guided with these templates are superposed to the canonical representatives of the inward and outward conformations. d) ALEPH identifies the largest differences between helices in both conformations (red).

**Table S3.** Predictions of the interactions building the *L. acidophilus* S-Layer

| Run | Input | Interaction | Result | Surface | ∆G Kcal/mol | Results | |  |
| --- | --- | --- | --- | --- | --- | --- | --- | --- |
| 1 | Native monomer | – | 5 equal models | – | – | Fig. 2a | |  |
| 2 | Tnaiveala, T8BT9ala, features.pkl, full msa | D2-D2 | 5 equal models | 571.4 | -6.9 | Fig. 2b | |  |
| 3 | Tnaiveala, T7QLEala, features.pkl full msa | D2-D2 | 4 equal models | 628.3 | -6.5 | As in Fig. 2b | |  |
| 4 | Tnaiveala, T7QLEala, features.pkl, msa10 | D1-D1 | 2 equal models | 409.3 | -0.3 | Fig. 2c | |  |
| 5 | Tnaiveala, T7QLEala, features.pkl, full msa msa_mask: 172-178, 212-220 | D1-D1 /  D1-D2 | Ranked 0-1  Ranked 3 | 413  698 | -0.4  -4.4 | As in Fig. 2c  As in Fig. 2d | |  |
| 6 | T7QLEala, features.pkl, fullmsa, msa_mask: 172-178, 212-220 | D1-D1 /  D1-D2 | Ranked 0-1  Ranked 2,4 | 430  694 | -1.0  -4.1 | As in Fig. 2c  Fig. 2d | |  |
| 7 | Nter tetramer: T7QLE, ranked_2 from run 6 polyala, features.pklfullmsa | D1-D1 +  D1-D2 | Ranked 0-1 | 596.3  457.7 | -4.7  2.2 | Fig. 2e | | |
| 8 | Cter tetramer: T8BT9, ranked_2 from run 6, features.pkl, fullmsa, msa_mask: 172-178, 212-220 | D1-D2 +  D2-D2 | Ranked 0-1 | 470  735 | -6.7  -4.2 | Fig. 2f |  |  |
| 9 | Mosaic hexamer fusing 7 and 8 | D1-D1 +  D1-D2 +  D2-D2 | Ranked 0 | 432  619  469 | 2  -4.2  -6.2 | Supplementary video 1 |  |  |

**
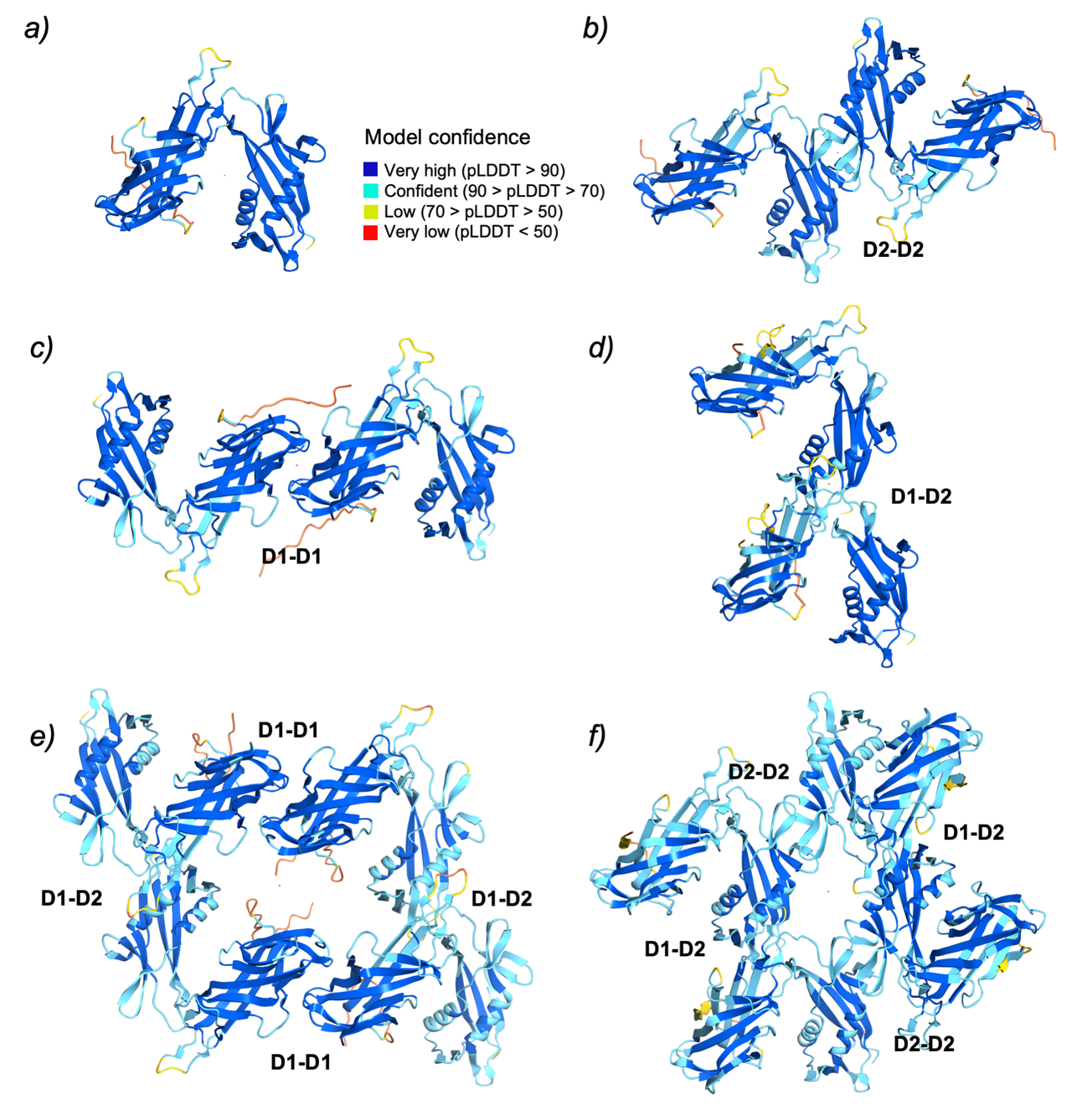
Figure S3.** Application of VAIRO to multimeric predictions within the *L. acidophilus* S-layer (SlpA); monomers are shown coloured by pLDDT values. a) Native AF prediction of the SLP monomer exhibiting only domains involved in the self-assembly (D1 and D2). b) SLP dimer associated through the D2 domains. c) SLP dimer associated through the D1 domains. d) SLP dimer associated through one D1 and one D2 domains. e) SLP tetramer associated through D1 domains and through one D1 and one D2 domains. f) SLP tetramer associated through D2 domains and through one D1 and one D2 domains.

**
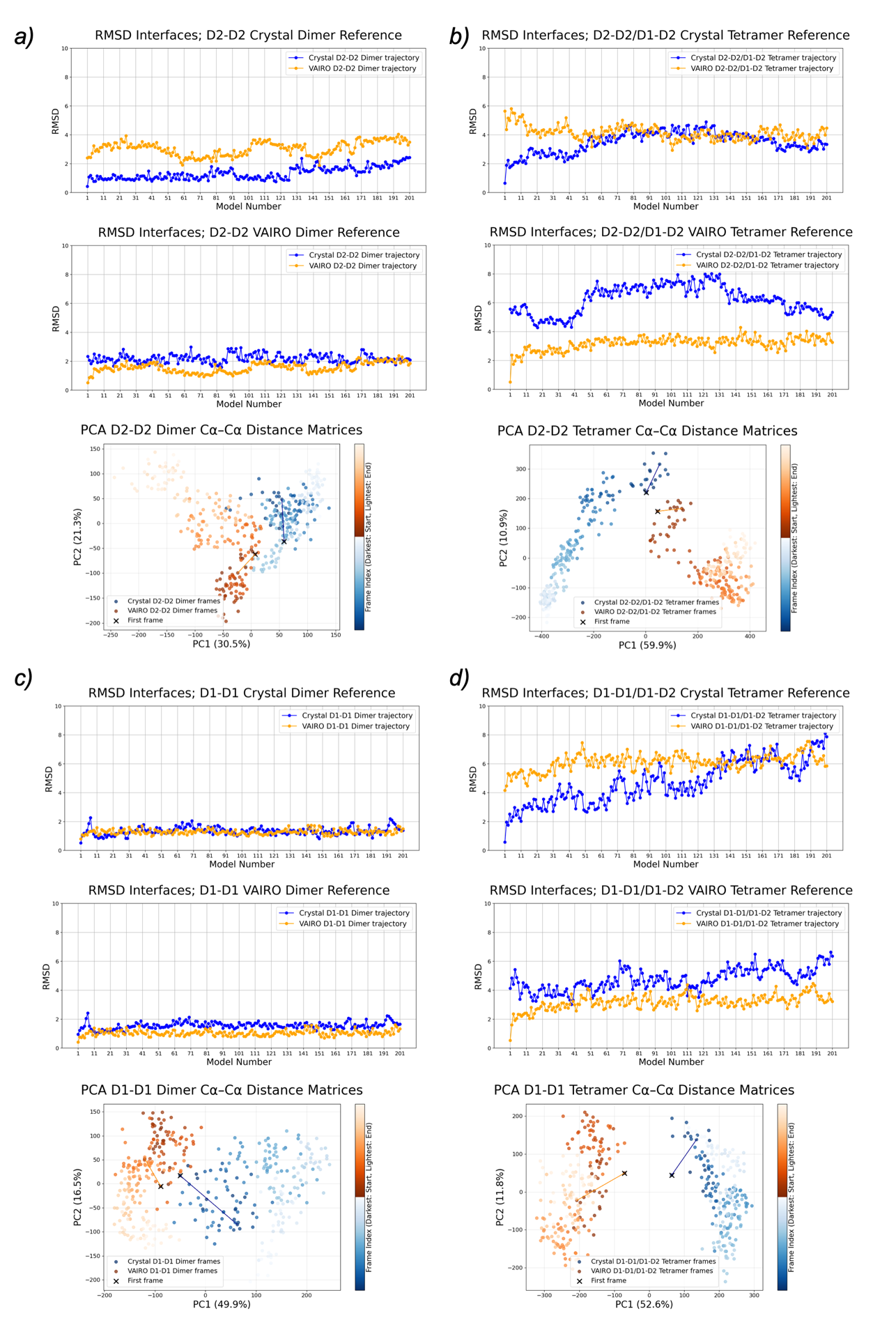
**

**Figure S4.** Analysis of the MD simulation trajectories for oligomers within the crystallographic structure (blue), and the corresponding predictions from VAIRO (orange). Cα rmsd between corresponding interfaces using the experimental structure as reference (top), the VAIRO model as reference (middle) and PCA for both (bottom). Solid colors mark the start of each trajectory and transparent ones the end. A cross marks the start of each trajectory, an arrow indicates the directions of the first ten frames and transparency of the colors increase along the trajectory. a) D2-D2 interface in dimer context b) D2-D2 bound tetramer containing D2-D2 and D1-D2 interactions. c) D1-D1 interface in dimer context. d) D1 bound tetramer containing D1-D1 and D1-D2 interactions. The plots reflect higher mobility around the D1-D2 interaction.


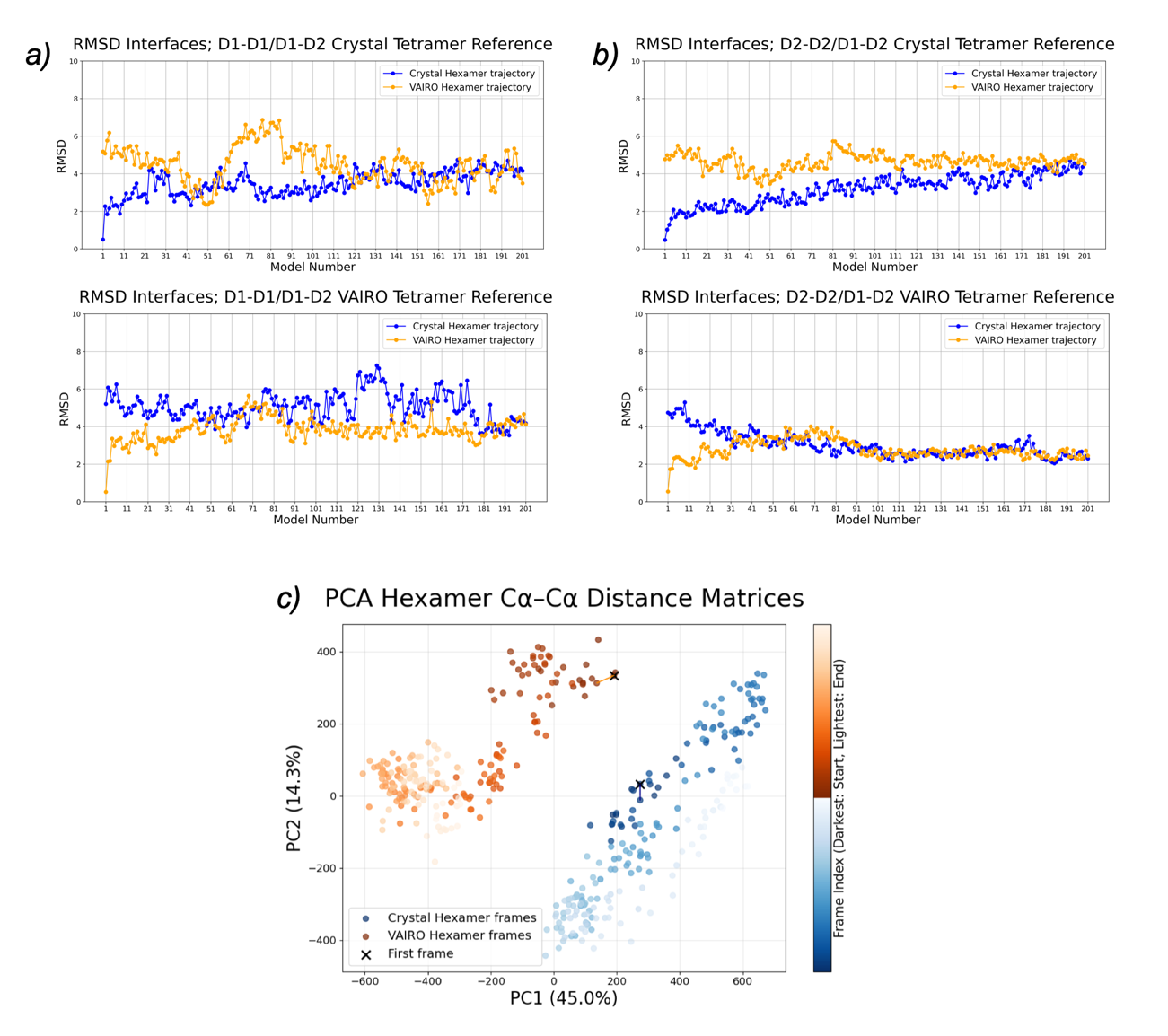


**Figure S5.** Analysis of the MD simulation trajectories for hexamers within the crystallographic structure (blue), and the corresponding prediction from VAIRO (orange). Cα rmsd between corresponding interface for the two alternative tetramers within the hexamer using the experimental structure as reference (top), the VAIRO model as reference (middle) and PCA for both (bottom). Solid colors mark the start of each trajectory and transparent ones the end. A cross marks the start of each trajectory, an arrow indicates the directions of the first ten frames and transparency of the colors increase along the trajectory. a) Cα rmsd calculated with D1 bound tetramer interface containing D1-D1 and D1-D2 interactions in hexamer context b) Cα rmsd calculated with D2 bound tetramer interface containing D2-D2 and D1-D2 interactions in hexamer context c) PCA relating the trajectories of experimental and predicted hexamer.


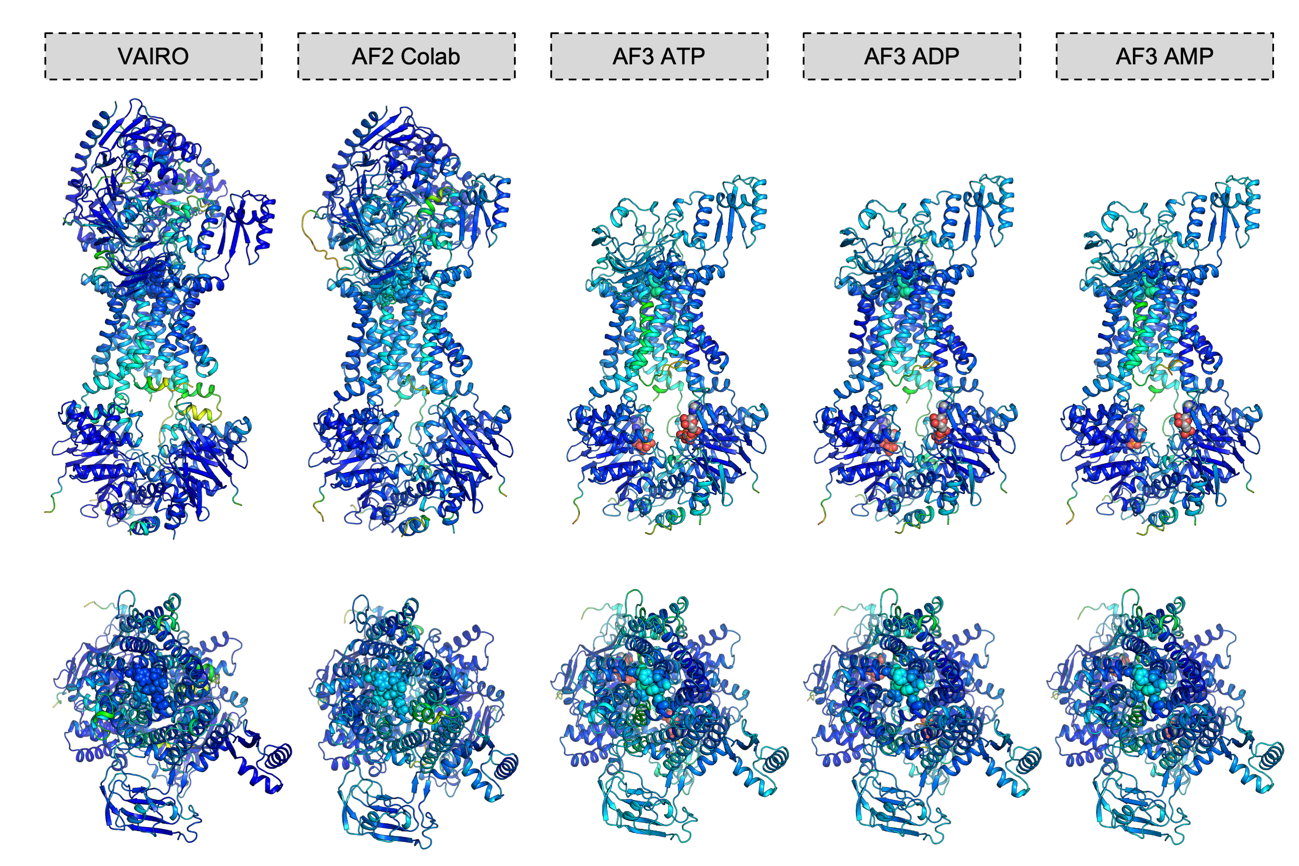


**Figure S6.** Inward-facing predictions of the Ami permease system through VAIRO in its mosaic mode, AlphaFold2 multimer obtained with ColabFold, and AlphaFold3 in complex with ATP, ADP and AMP. (§ Application to the Ami permease system from *Streptococcus pneumoniae*: clustering and direction from inward-facing towards outward-facing conformation)

**METHODS**

**Modes of use of VAIRO**

With minimum input-sequence information for a monomer or multimer, the **naïve mode**, an analysis of the native AF search for templates and sequences related to the target may be requested. Up to twenty templates are retrieved and structurally compared and if these can be divided into two or more consistent groups, these are segregated into individual processes, with the aim of retrieving predictions to represent each of the different structural groups seen in experimental structures. These extremes may encompass structural variations ranging from domain motions to secondary structure differences or small local changes.

Predictions following different structural models can also be explicitly controlled and compared in an extension of the naïve mode towards the **guided mode**, which requires external templates to be provided by the user. This mode bypasses the AF search for information to base the prediction on the models and sequences provided. Templates are PDB models or PDB codes for which a model can be retrieved, potentially expanded by symmetry operations to generate the annotated multimers. Directions can be given for templates to match a specific position(s) within the query sequence, and thus override its automatic placement through alignment and consistent matching to a previous reference template. Overall or partial modification of the template sequence can be requested. A pre-aligned sequence may be provided through an external file to define the modification of a template or to be incorporated into the MSA. Information from another prediction can be input through a previously generated pre-formatted serialized file (features.pkl, generated by AF to store all information specific to the query sequence), provided it was set up to predict the same or a formally equivalent structure (e.g., a chain of the same dimensions, conceivably within another multimer or with point mutations). Modifications can be requested to reduce the length and thus weight of the MSA or to erase the fields within selected regions. It can also be supplemented with additional lines or a library from which to extract sequences. A **mutate mode** further allows to introduce substitutions in the query sequence after the features.pkl has been set up. This operation may help avoiding rogue contacts, for instance to exposed hydrophobic transmembrane residues, or shield interactions in the resulting prediction. It triggers a second prediction in which the mutation is reverted.

The **mosaic mode** divides sequences too large for the available hardware and may be requested by stating the number of partial overlapping structures to predict and blend. It may be combined with other modes.

**Input**

The query sequence to be predicted establishes the reference upon which all actions are directed and any item input must refer to an amino acid in this sequence. For a multimer, one or more copies of the sequence(s) provided are concatenated with glycine stretches as linkers. The length of the linkers does not appear to be critical in our tests and unless otherwise specified will default to 50. Predicted models are calculated in the monomeric mode of AF2, with the assembled sequence setting the dimension of the features.pkl array, which holds all the information used by AF beyond the learned weights. Hence, further comparisons can all be referred to the common alignment, which is implicitly enforced in the prediction.

Templates are automatically directed into the features array after extracting their sequence and aligning it against the query sequence with the HHsuite software (Steinegger et al., 2019). When homomultimeric templates are input, sequence alone would entail ambiguity in the assignment, potentially leading to inconsistent information in the case of asymmetric multimeric structures. Therefore, the automatic procedure will optimize the structural match to a reference template, which can be specified in the input or is otherwise selected as the first one given. If the relevant multimer is not present in the asymmetric unit of the template, application of symmetry from the biological entity annotation can be requested in order to build it. Pre-aligned templates, for instance from a previous run or manually annotated, can be input and directed to a position in the multimer, where they will match those amino acids with equal sequence numbers. Libraries of superimposed peptide fragments need to be directed to a common position as in general they are too short for the alignment algorithms.

Template sequence has been found to influence its weight on the prediction. To this end, sequence modifications specified in the input are performed after template alignment. Adopting the query sequence is used to upweight the information implicit in a template, e.g., to protect the environment of an active site or ligand. On the contrary, modifying the sequence to polyalanine will allow larger deviations in the predicted model. Sequence modification can be directed by specifying selected positions, giving overall changes in a range to modify all amino acids to e.g., serine or matched to any specified sequence in a file. Template information is ultimately introduced in the features.pkl file minding the gaps in the numbering to match the polyglycine stretches inserted in the sequence.

Additional sequences may be input into the MSA through PDB files, whether they are used as templates or not, or through ALEPH (Sammito et al., 2013) libraries to enforce contact interactions.

Controlled changes in the sequence to be predicted that are reverted prior to the relaxation step can be performed. We name them mutations, in analogy to the ones that would be experimentally performed to abolish an interaction relevant for function. Mutation should be used with care.

Structural comparisons are required at several stages in the method: to compare templates in a naïve run and partition the set into clusters, to ensure consistency among different multimeric templates when the particle formed is asymmetric, and to interpret the resulting predictions analyzing their similarity to templates and experimental references, if provided. In a naïve run, the features.pkl array is divided after template analysis to represent the resulting clusters and the predictions proceed independently. In the case of sequences too large for the GPU, the features.pkl is also divided but into overlapping sections of the assembled sequence. The fragments are subsequently fused into a prediction using LSQKAB. For multimers, the interfaces formed are also compared and analyzed. Their energy as estimated with PISA/OpenMM is consulted to discard unrealistic predictions.

**Analysis of the structural context: HINGES**

Preliminary questions in structure comparison are whether enough data are available to support the intended purpose and the extent of the differences under consideration. We check whether the templates share sufficient identity and coverage to be compared, that Ramachandran outliers are less than 5%. Assessing if differences involve domain motions, changes in orientation of secondary structure elements, or more subtle local variations is achieved by successive superposition of rigid groups after optimizing the points at which they should be divided in both structures under comparison.

The first comparison, which gives more detailed information about differences between structural models and does not require more than two models, was implemented in HINGES. This program conceptually first superimposes all equivalent partial residue stretches of two structures and stores the SSQ (sum of squares) for each superposition. Based on this table, it determines the residue that yields the lowest SSQ_tot_ = SSQ_before_ + SSQ_after_ for the separate superposition of pairs of equivalent residues from the two structures before and including its position (SSQ_before_), and after it (SSQ_after_). This residue is considered as a hinge, and the hinge angle is calculated. The procedure is recursively repeated for a higher number of hinges. Since introducing another hinge means raising the number of degrees of freedom, the SSQ_tot_ decreases with the number of hinges. The behavior of the decrease and its asymptotic value depends on the actual amount and complexity of differences in conformation of the two structures, and on the precision of their coordinates.

The match of residues to be superposed is derived from their previous alignment in the features.pkl array and all possible pairs of corresponding residues are included in the calculation. The first rmsd value before hinges are introduced gives an idea of the magnitude of the structural differences: analyzing a table of pairwise comparisons may reveal consistent subsets, which are validated by sequence identity towards the query sequence. Conversely, once a rigid group responsible for large differences is identified, the same partition can be tested for all models. The extent of the differences allows to differentiate between domain motions, secondary structure motions and local differences. The hypothesis is confirmed when the structures become equivalent after a number of subdivisions. If templates overlap by less than 60% of their chain in the multimer or dividing into rigid groups does not lead to a low rmsd, automatic comparison is not possible.

**Analysis of structural groups with PDB2CC and CCANALYSIS**

A second classification step uses a novel algorithm for unsupervised learning, termed CCANALYSIS (Brehm & Diederichs, 2014; Diederichs, 2017). CCANALYSIS is agnostic of the actual data. For a 2-dimensional analysis, it only requires as input the pairwise similarities between at least 5 objects. CCANALYSIS can be used for e.g., sequence comparisons (Fleming, Muller, Zacharchenko, Diederichs, & Mayans, 2023; Su, Mayans, Diederichs, & Fleming, 2022), image comparisons (Diederichs, 2017) or resolving an indexing ambiguity in crystallographic data (Brehm & Diederichs, 2014; White et al., 2016). The mathematical requirement for its use is that the pairwise similarities can be estimated in a similar way to a Pearson correlation coefficient (Hunkler, Diederichs, Kukharenko, & Peter, 2023). This is fulfilled if the similarity of two objects that differ only by random noise from each other and a third object equals the product of the similarity values obtained for each of the two objects against the third object.

For structure comparison, the similarity of any pair of models is estimated by PDB2CC. PDB2CC optionally calculates the correlation between intra-molecular distance sets. For the analyses presented here, a different option is used: for each pair of superimposed Cα atoms, the program calculates the value $e^{\frac{-x^{2}}{2}}$, where x is the distance between the atoms. These values are used as weights in iterated superpositions, and their average is used as the numerical estimate of the structural similarity. It can be shown that this similarity measure fulfills the requirement for the applicability of CCANALYSIS.

The PDB2CC-CCANALYSIS method arranges structural models in the plane of a circle with radius 1, such that systematic structural differences, e.g., grouped changes of conformation as those arising in allosteric enzymes upon binding of a ligand, are revealed through directional differences between vectors that represent the models. Large structural differences between models correspond to angles between vectors up to 90 degrees; absence of systematic structural differences results in vectors pointing in the same direction. If more than one mode of systematic conformational change should be analyzed, the analysis can be performed in a 3-dimensional sphere instead of a 2-dimensional circle; even higher dimensionality is possible but difficult to visualize. For the work reported here, we adopted the 2-dimensional analysis.

Random structural differences between models (such as those arising from differences in experiment or refinement) determine the vector lengths. The maximum vector length of 1 corresponds to prototypical (ideal) protein models, whereas a vector length approaching 0 would represent increasingly strong but non-systematic deviations from other models whose vectors point in the same direction. The quantitative relationship between length and amount of randomness is given in (Diederichs, 2017).

The 2-dimensional separation of differences into those arising from systematic differences, which are partially shared between models, and random differences, which are unique to pairs of structural models, allows a meaningful clustering of models if required, e.g., with routines such as k-means. For our purposes of identifying extreme conformations, we use the (one-dimensional) angle assigned to each model in a 2-dimensional analysis.

Other pipelines for comparing protein conformational states are available and might provide future complementary criteria (Ellaway et al., 2023).

**Connecting overall differences with most relevant local variations with ALEPH difference matrices**

Local analysis to identify regions showing main differences in the templates is also performed in ALEPH (Medina et al., 2019) whereby difference matrices on properties abstracting the backbone geometry are computed between models. Aligned reference structures, templates and the predicted structures produced at different stages by AF can be annotated through a series of specialized Characteristic Vectors (CV) (Sammito et al., 2013) that can simultaneously describe secondary and tertiary structures properties of the fragments. Each CV is computed from a pre-determined number of residues with a minimum of tripeptides. Therefore, matrices of spatial relationships (angles and distances among vectors) can be computed from any given PDB and comparisons are possible without performing superposition. After the normalization of such matrices, based on capped distances and maximum angles admitted, a direct matrix distance metric is used to establish their similarities. We employ Frobenius norm (Golub, 1996) to generate a scalar to measure fold similarities.

$\left\| A \right\|_{F}=\sqrt{Tr\left( {AA}^{H} \right)}$ *(1)*

the square root of the matrix trace of AA^(H), where A^(H) is the conjugate transpose. The Frobenius scores among templates and predicted structures provide an alternative way to divide templates into groups by hierarchical clustering displayed as a dendogram, although by default we rely on CCANALYSIS for this task.

To connect the overall differences reduced to a single score with identification of the largest local differences in the analysis we generate matrices from different CVs sizes (one vector for each secondary structure element) calculate cumulative angle differences for each of these elements to all others and provide a PyMOL (Schrödinger, 2015) session with superimposed predictions and reference structures where states highlighting the largest deviations have been annotated and can be sequentially visited for examination in the graphics.

**Analysis of predictions**

The structural soundness of the resulting predictions is established by filtering out misfolded models consulting the average pLDDT, and the geometry by Ramachandran outliers and compactness, calculated as the average number of residues within a radius of 10 Å excluding linked residues. Predicted models are superposed with each other, the templates used and the omitted ones, and reference structures if available with GESAMT from the CCP4 suite (Agirre et al., 2023). Again, CCANALYSIS and differences in the distribution of characteristic vectors, implemented in ALEPH, are used to characterize and quantify changes in conformations and interactions. PISA is used to identify interface regions when a multimer is predicted. Energy evaluation in PISA and/or OpenMM, is used to assess the plausibility of the structures and whether the interactions induced are likely to occur.

**Output**

The standard output of the VAIRO run is presented in an HTML format file, structured into several sections. The predicted models are embedded in the HTML and can be downloaded or displayed from a PyMOL session, a preset series of views showing superpositions of predictions, templates and references allowing to display main changes and similarities and regions of interest. The report begins with a summary of the mode and results, followed by a comprehensive record in a command file of the input parameters used, for future reproducibility.

The alignment graph (for an example see Fig. 6a) allows to apprehend at a glance the information input and modifications requested. The sequence(s) to be predicted are at the base of the plot, separated by linkers and sequentially numbered, mutations of this sequence are marked in red. On top of it, templates are aligned at the position where they are input, showing the original sequence similarity to the target through a scale of gray (black for identical and light for dissimilar) and modifications in the sequence with which they are entered into the features.pkl array are highlighted in red for an external file, typically the target FASTA sequence to enhance their weight or yellow for polyalanine to depress it. In the case of libraries, templates may be too numerous to show. A maximum of 20 are sampled and an average is summarized. For the MSA, given its extension average coverage is displayed in a single line. Masking of the MSA is shown in white. Subsequently, the plot of pLDDT displays the predicted LDDT per residue position for all predictions.

The clustering results from CCANALYSIS are also included, capturing main structural differences, e.g., different conformations between templates and predictions (if any). The interface analysis section is included only if a multimer is predicted, this includes solvation energy estimates for each association and the templates where the interfaces can be found.

The output contains further tables: the average pLDDT for each prediction; Ramachandran outliers and compactness; percentages of secondary structure elements calculated with ALEPH; estimated energies for the predictions measured in (Kcal/mol); superposition between predictions; superposition between predictions and templates; the superposition between reference PDB files with predictions and templates, if experimental structures are given. All superpositions are quantified by rmsd values. And finally, the output includes the logfile generated during the run, and conclusions to highlight the most relevant results.

**Computing requirements**

Predictions of Selecase and immunoglobulin were performed on a 24-core workstation with Intel Core i9-12900KF and 64 GB RAM, an NVIDIA GA102 GeForce RTX 3090 graphics card with 24 GB memory running Debian GNU/Linux 11; runtimes ranged from 5 to 13 minutes. Predictions of the transmembrane dimer of the Ami permease system took 90 minutes on a 48-core workstation with Intel Xeon Gold 6136 and 128 GB RAM, an NVIDIA PNY Quadro RTX A6000 graphics card with 48 GB memory, running Debian GNU/Linux 9.

**Molecular dynamics (MD) simulations**

MD simulations were performed with GROMACS version 2024.2 (Hess et al., 2008), using OPLS-AA/L all-atom force field (Jorgensen, Maxwell, & Tirado-Rives, 1996). The oligomers were placed in a dodecahedron box leaving 20 Å to the edge of the box and solvated with spce (SPC/E) water molecules (Chatterjee, Debenedetti, Stillinger, & Lynden-Bell, 2008). Long-range electrostatic interactions were treated using the particle mesh Ewald method (Darden, York, & Pedersen, 1993; Essmann et al., 1995).

A first minimization step of 5000 steps using steepest descent method was followed by 5000 steps of conjugate gradient algorithm. Heating phase was performed with the modified Berendsen (Bussi, Donadio, & Parrinello, 2007) thermostat until reaching 300K. The last equilibration phase was performed using the C-rescale barostat (Bernetti & Bussi, 2020) at 1 atm. Finally, two replicas of production runs were performed for each oligomer, reaching a total simulation time of 100ns. Hydrogen bonds were constrained during both equilibration and production runs using holonomic constraints.

**REFERENCES**

Agirre, J., Atanasova, M., Bagdonas, H., Ballard, C. B., Basle, A., Beilsten-Edmands, J., … Yamashita, K. (2023). The CCP4 suite: Integrative software for macromolecular crystallography. *Acta Crystallogr D*, *79*(Pt 6), 449–461. doi: 10.1107/S2059798323003595

Bavnhoj, L., Paulsen, P. A., Flores-Canales, J. C., Schiott, B., & Pedersen, B. P. (2021). Molecular mechanism of sugar transport in plants unveiled by structures of glucose/H(+) symporter STP10. *Nature Plants*, *7*(10), 1409–1419. doi: 10.1038/s41477-021-00992-0

Bernetti, M., & Bussi, G. (2020). Pressure control using stochastic cell rescaling. *The Journal of Chemical Physics*, *153*(11), 114107. doi: 10.1063/5.0020514

Brehm, W., & Diederichs, K. (2014). Breaking the indexing ambiguity in serial crystallography. *Acta Crystallographica*, *D70*(Pt 1), 101–109. doi: 10.1107/S1399004713025431

Bussi, G., Donadio, D., & Parrinello, M. (2007). Canonical sampling through velocity rescaling. *The Journal of Chemical Physics*, *126*(1), 014101. doi: 10.1063/1.2408420

Chatterjee, S., Debenedetti, P. G., Stillinger, F. H., & Lynden-Bell, R. M. (2008). A computational investigation of thermodynamics, structure, dynamics and solvation behavior in modified water models. *The Journal of Chemical Physics*, *128*(12), 124511. doi: 10.1063/1.2841127

Darden, T., York, D., & Pedersen, L. (1993). Particle mesh Ewald: An N⋅log(N) method for Ewald sums in large systems. *The Journal of Chemical Physics*, *98*(12), 10089–10092. doi: 10.1063/1.464397

Diederichs, K. (2017). Dissecting random and systematic differences between noisy composite data sets. *Acta Crystallographica*, *D73*(Pt 4), 286–293. doi: 10.1107/S2059798317000699

Ellaway, J. I. J., Anyango, S., Nair, S., Zaki, H. A., Nadzirin, N., Powell, H. R., … Velankar, S. (2023). Automated Pipeline for Comparing Protein Conformational States in the PDB to AlphaFold2 Predictions. *bioRxiv*, 2023.07.13.545008. doi: 10.1101/2023.07.13.545008

Essmann, U., Perera, L., Berkowitz, M. L., Darden, T., Lee, H., & Pedersen, L. G. (1995). A smooth particle mesh Ewald method. *The Journal of Chemical Physics*, *103*(19), 8577–8593. doi: 10.1063/1.470117

Fleming, J. R., Muller, I., Zacharchenko, T., Diederichs, K., & Mayans, O. (2023). Molecular insights into titin’s A-band. *Journal of Muscle Research and Cell Motility*. doi: 10.1007/s10974-023-09649-1

Golub, G. H. and V. L. (1996). *Matrix Computations* (Third). The Johns Hopkins University Press.

Hess, B., Kutzner, C., van der Spoel, D., & Lindahl, E. (2008). GROMACS 4: Algorithms for Highly Efficient, Load-Balanced, and Scalable Molecular Simulation. *Journal of Chemical Theory and Computation*, *4*(3), 435–447. doi: 10.1021/ct700301q

Hunkler, S., Diederichs, K., Kukharenko, O., & Peter, C. (2023). Fast conformational clustering of extensive molecular dynamics simulation data. *Journal of Chemical Physics*, *158*(14), 144109. doi: 10.1063/5.0142797

Jorgensen, W. L., Maxwell, D. S., & Tirado-Rives, J. (1996). Development and Testing of the OPLS All-Atom Force Field on Conformational Energetics and Properties of Organic Liquids. *Journal of the American Chemical Society*, *118*(45), 11225–11236. doi: 10.1021/ja9621760

Medina, A., Triviño, J., Borges, R. J., Millán, C., Usón, I., & Sammito, M. (2019). ALEPH: a network-oriented approach for the generation of fragment-based libraries and for structure interpretation. *Acta Crystallographica*, *D76*.

Sammito, M., Millán, C., Rodríguez, D. D., de Ilarduya, I. M., Meindl, K., De Marino, I., … Usón, I. (2013). Exploiting tertiary structure through local folds for crystallographic phasing. *Nature Methods*, *10*(11), 1099–1101. doi: 10.1038/nmeth.2644 http://www.nature.com/nmeth/journal/v10/n11/abs/nmeth.2644.html#supplementary-information

Schrödinger. (2015). *The PyMOL Molecular Graphics System, Version 1.8*.

Steinegger, M., Meier, M., Mirdita, M., Vohringer, H., Haunsberger, S. J., & Soding, J. (2019). HH-suite3 for fast remote homology detection and deep protein annotation. *BMC Bioinformatics*, *20*(1), 473. doi: 10.1186/s12859-019-3019-7

Su, K., Mayans, O., Diederichs, K., & Fleming, J. R. (2022). Pairwise sequence similarity mapping with PaSiMap: Reclassification of immunoglobulin domains from titin as case study. *Computational and Structural Biotechnology Journal*, *20*, 5409–5419. doi: 10.1016/j.csbj.2022.09.034

White, T. A., Mariani, V., Brehm, W., Yefanov, O., Barty, A., Beyerlein, K. R., … Chapman, H. N. (2016). Recent developments in CrystFEL. *Journal of Applied Crystallography*, *49*(Pt 2), 680–689. doi: 10.1107/S1600576716004751
